# Natural variation in a *FtMYB32* ‐ *FtCES18* module drives *Rhizoctonia solani* resistance by reprogramming jasmonate and flavonoid metabolism in Tartary buckwheat

**DOI:** 10.1111/tpj.71176

**Published:** 2026-10-26

**Authors:** Yuanyuan Chen, Yang Liu, Xixu Peng, Haihua Wang, Zhengwu Fang, Jai Chand Rana, Nikhil Chrungoo, Maria D Logachev, Chaonan Guan, Meiliang Zhou

**Affiliations:** ^1^ Sanya Nan Fan Research Institute of Chinese Academy of Agricultural Sciences Sanya Hainan 572024 China; ^2^ Hainan Seed Industry Laboratory Sanya 572024 China; ^3^ State Key Laboratory of Crop Gene Resources and Breeding, Institute of Crop Sciences Chinese Academy of Agricultural Sciences Beijing 100081 China; ^4^ College of Agriculture Yangtze University Jingzhou Hubei 434023 China; ^5^ School of Life and Health Sciences Hunan University of Science and Technology Xiangtan Hunan 411201 China; ^6^ Alliance of Bioversity International and CIAT New Delhi India; ^7^ School of Life Sciences Royal Global University Guwahati India; ^8^ Skolkovo Institute of Science and Technology Moscow Russia; ^9^ Vavilov Institute of General Genetics Russian Academy of Science Moscow Russia; ^10^ Institute of Economic Crops Henan Academy of Agricultural Sciences Zhengzhou 450003 China

**Keywords:** Tartary buckwheat, *Rhizoctonia solani*, *FtCES18*, *FtMYB32*, natural variation, JA, flavonoids

## Abstract

Tartary buckwheat *(Fagopyrum tataricum*) is renowned for its adaptability to complex growing conditions and stress tolerance. However, soil‐borne necrotrophic pathogens such as *Rhizoctonia solani* frequently cause devastating diseases that severely compromise its yield and quality. In this study, through a genome‐wide association study (GWAS), we identified a carboxylesterase gene, *FtCES18*, and demonstrated that it acts as a core positive regulator of disease resistance in Tartary buckwheat. Overexpression of *FtCES18* significantly enhanced plant resistance, whereas virus‐induced gene silencing (VIGS) led to a highly susceptible phenotype. Further analyses revealed that FtCES18 efficiently catalyzes the hydrolysis of methyl jasmonate (MeJA) into free jasmonic acid (JA), which initiates downstream JA‐Ile signaling and rapidly promotes endogenous jasmonate accumulation upon pathogen infection. This JA signaling cascade reprograms host flavonoid metabolism, promoting the substantial accumulation of downstream defensive flavonoids (such as rutin and quercetin). Furthermore, we found that the transcription factor FtMYB32 directly binds to the MBS cis‐element in the *FtCES18* promoter to activate its expression. Notably, evolutionary analysis revealed a domestication‐associated C‐terminal truncation in the cultivated FtMYB32 that impairs its transactivation capacity, explaining the compromised basal resistance in cultivated accessions compared to their wild ancestors. Crucially, a natural single‐nucleotide polymorphism (SNP) in the *FtMYB32* promoter defines a superior haplotype (hapC) conferring enhanced basal transcriptional activity. This natural variation in promoter activity controls the expression abundance of the *FtMYB32*‐*FtCES18* module via a “dosage fine‐tuning” effect, which is subsequently amplified by the downstream metabolic cascade, ultimately contributing to the natural variation in disease resistance among different Tartary buckwheat accessions. This study provides valuable genetic resources and a theoretical basis for breeding novel Tartary buckwheat varieties with high disease resistance.

## Significance Statement

This study uncovers a genetic and metabolic mechanism linking natural regulatory variation to disease resistance in Tartary buckwheat. FtMYB32 activates FtCES18‐mediated conversion of MeJA to JA, thereby enhancing jasmonate signaling and redirecting flavonoid metabolism toward defensive compounds, while a favorable *FtMYB32* promoter haplotype strengthens this pathway and offers a potential target for resistance breeding.

## INTRODUCTION

Tartary buckwheat (*Fagopyrum tataricum* (L.) Gaertn.) is an important pseudocereal crop renowned for its exceptional nutritional value and health‐promoting benefits (H. Zhao, He, et al., 2023), which are largely attributed to its abundance of flavonoid secondary metabolites, such as rutin, that are highly effective in preventing human cardiovascular diseases (Lai et al., 2024; Wang et al., 2025). However, the agricultural production and seed quality of Tartary buckwheat are severely threatened by various fungal diseases (He et al., 2023). Among them, root rot and damping‐off caused by *Rhizoctonia solani* are highly destructive soil‐borne diseases (Wibberg et al., 2013; Zheng et al., 2013). Although *R. solani* is primarily classified as a soil‐borne pathogen, it is also capable of developing aerial mycelia to infect other aboveground tissues. *R. solani* not only causes substantial yield losses or even total crop failure in Tartary buckwheat fields but also inflicts devastating diseases worldwide on various major economic crops, including rice, maize, and wheat (Li et al., 2019; Okubara et al., 2014; Smiley, 2021; Zhang et al., 2021). Currently, field management of this disease relies heavily on chemical fungicides, whereas cultural practices and biological controls offer limited efficacy (Molla et al., 2020). Consequently, identifying endogenous disease‐resistance genes and breeding resistant varieties represent the most economical, effective, and environmentally friendly strategies for controlling this disease.

Plant defense against pathogen attack typically involves the activation of phytohormone signaling pathways, including salicylic acid (SA), jasmonic acid (JA), and ethylene (ET) (Bari & Jones, 2009). Specifically, against necrotrophic fungi such as *R. solani*, the JA pathway serves as the central signaling hub that drives defensive transcriptional reprogramming and the accumulation of secondary metabolites (Navarro et al., 2008). Within the landscape of JA metabolic homeostasis, MeJA functions primarily as a highly volatile and relatively bio‐inactive storage pool. Upon perceiving pathogen invasion, plants must rapidly demethylate (hydrolyze) the accumulated MeJA into free JA, which is subsequently conjugated to form the ultimate active signaling molecule, JA‐Ile, in order to activate the downstream defense signaling cascade (Kneeshaw et al., 2022; Wasternack & Song, 2017). This hydrolytic conversion represents a critical rate‐limiting step governing the amplitude of JA signal output and is specifically catalyzed by carboxylesterases (CES) or methylesterases (MES) possessing an α/β‐hydrolase fold domain (Cao et al., 2019; Xiao et al., 2024). For instance, in wild tobacco (*Nicotiana attenuata*), silencing the methyl jasmonate esterase gene (*NaMJE*) substantially impairs the conversion of MeJA to JA, which deprives the plants of the ability to initiate an endogenous JA burst and subsequent defense gene expression upon external biotic stress, thereby rendering them highly susceptible (Wu et al., 2008). Similarly, in *Arabidopsis thaliana*, several members of the MES family (e.g., *AtMES10*) have been confirmed to possess specific MeJA hydrolytic activity, playing an indispensable role in fine‐tuning the dynamic homeostasis of the active JA pool during plant stress responses (Yang et al., 2008). Nevertheless, the specific biological functions and substrate catalytic properties of CES family proteins in Tartary buckwheat, particularly in response to soil‐borne fungal stress, remain largely uncharacterized.

Beyond hormone signaling networks, the accumulation of defensive secondary metabolites constitutes another critical line of defense in the plant immune system (Li et al., 2025; Su et al., 2025). As a distinctive medicinal and edible crop, Tartary buckwheat is rich in flavonoids, typically represented by rutin and quercetin (Kirschner, 2022; Li et al., 2021). Flavonoids play a crucial role as phytoalexins and antioxidants in plant defense against fungal pathogen infection (Shen et al., 2022; Treutter, 2005). For instance, quercetin can directly inhibit the mycelial growth of the pathogenic fungus *Phytophthora nicotianae* (Duan et al., 2025), and it can also mitigate host cell necrotic damage by scavenging excessive reactive oxygen species (ROS) induced by pathogen infection (Agati et al., 2012). The biosynthesis of these defensive flavonoids is governed by strict transcriptional reprogramming within the plant. Among numerous regulatory factors, the MYB transcription factor family acts as a “master switch” in integrating environmental stimuli and orchestrating secondary metabolic networks (Ding et al., 2021; Jiang et al., 2023; Matsui et al., 2018). MYB transcription factors can specifically recognize and bind to *cis*‐acting elements (e.g., MBS elements) within the promoters of downstream target genes (Jiang et al., 2022), thereby precisely regulating flavonoid metabolism and broad‐spectrum disease resistance in plants. However, it remains largely elusive which core MYB transcription factors participate in this complex defensive network in Tartary buckwheat and coordinate the expression of the downstream carboxylesterase.

In this study, by combining GWAS based on the disease indices of 320 Tartary buckwheat accessions with temporal transcriptomics, we identified a core carboxylesterase gene, *FtCES18*, as a key regulator of resistance against *R. solani*. *In vitro* and *in vivo* functional characterizations demonstrated that FtCES18 coordinately activates JA signaling and enhances flavonoid biosynthesis while bolstering the host antioxidant defense machinery to eliminate excessive ROS, which together contribute to significantly improved plant disease resistance. Exploring its upstream regulatory network, we identified a core transcription factor, FtMYB32, and confirmed that it directly binds to the MBS elements within the *FtCES18* promoter to positively activate its expression. Moreover, comparative analysis between wild and cultivated accessions revealed a domestication‐associated C‐terminal truncation in cultivated FtMYB32 that significantly attenuates its transactivation efficacy. More importantly, we discovered that a natural variation (a T/C single nucleotide polymorphism) in the *FtMYB32* promoter serves as the crucial genetic basis driving the divergence of disease resistance among the natural Tartary buckwheat populations. Accessions harboring the favorable haplotype hapC exhibit stronger basal and activated transcriptional activity of *FtMYB32*, thereby more effectively promoting the downstream “*FtCES18*‐JA‐flavonoid” defense cascade. This study not only elucidates the molecular and genetic mechanisms underlying Tartary buckwheat defense against necrotrophic soil‐borne pathogens but also provides clear theoretical insights and potential genetic marker targets for breeding highly disease‐resistant cultivars.

## RESULTS

### GWAS and transcriptomics identify a carboxylesterase gene associated with *R. solani* resistance

To evaluate the resistance of Tartary buckwheat germplasm to *R. solani*, we conducted disease resistance phenotyping on 320 core accessions previously established by our group (He et al., 2023). The phenotypic values exhibited continuous variation, which indicates the reliability of the phenotypic evaluation process and suggests that the disease resistance in this population is a complex quantitative trait controlled by multiple genes. Integrating previously published whole‐genome resequencing data of these core accessions (Zhang et al., 2021), we performed a GWAS to identify SNPs associated with *R. solani* resistance. The results revealed a significant association signal (‐log_10_ *P* > 5.0) on Chromosome 8 (Figure 1A). Linkage disequilibrium (LD) analysis (Figure 1B) showed a distinct strong linkage block within this candidate region, with the distribution of high R^2^ values indicating a strong correlation between the peak SNP and the surrounding variants.

**Figure 1:**
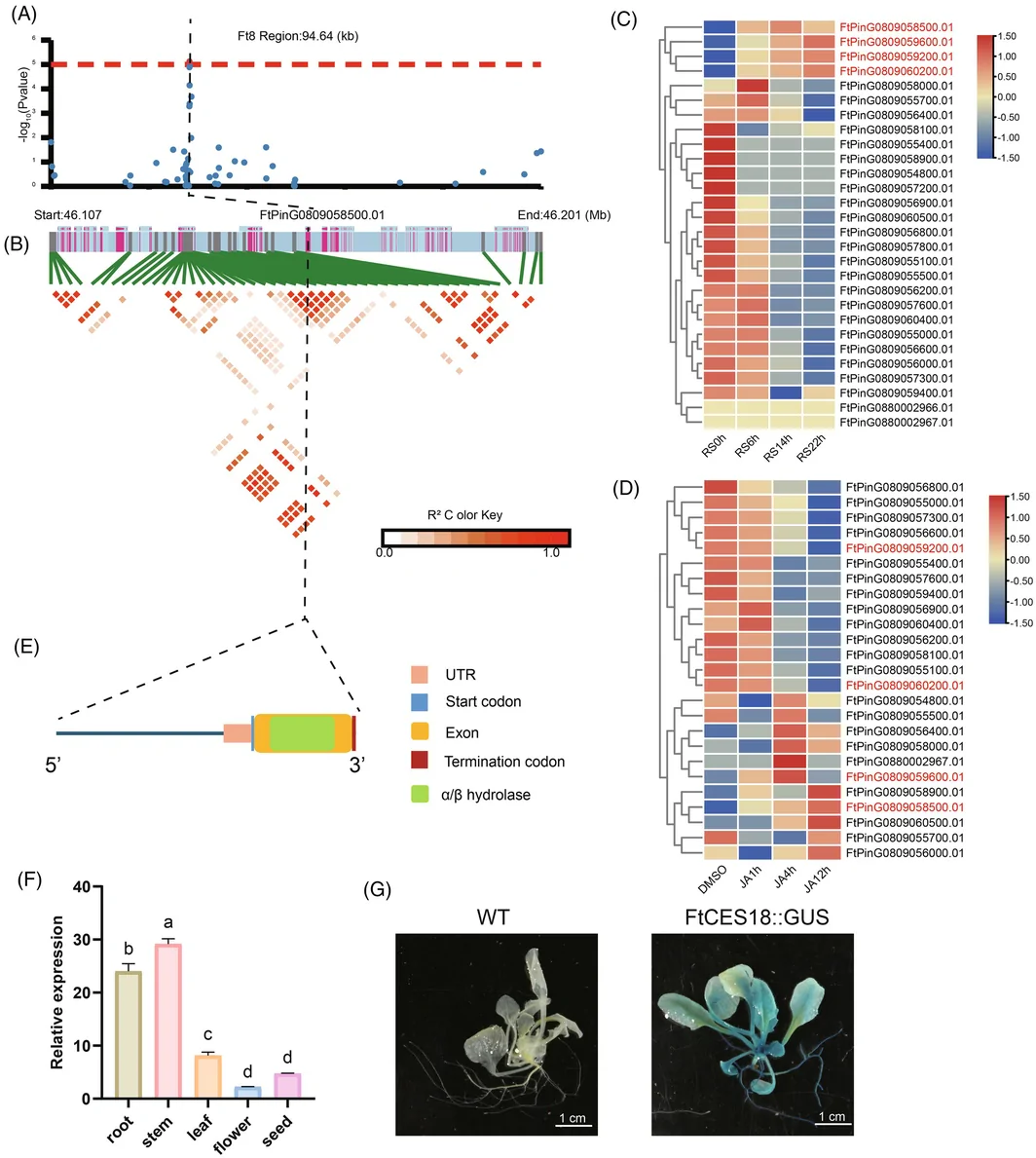
Identification and expression analysis of the candidate resistance gene *FtCES18*. (A) Local Manhattan plot for *R. solani* resistance on chromosome Ft8. The red dashed line indicates the significance threshold. (B) Linkage disequilibrium (LD) block spanning the 94.64‐kb significant region. Dashed lines indicate the physical position of FtPinG0809058500.01 (*FtCES18*). (C, D) Expression heatmaps of candidate genes in the LD block after *R. solani* inoculation (C) and exogenous JA treatment (D). The red boxes highlight *FtCES18*. (E) Gene structure and predicted conserved domains of *FtCES18*. The α/β‐hydrolase domain is shown in green. (F) Tissue‐specific expression of *FtCES18* in Tartary buckwheat. Data are shown as mean ± SD. Different lowercase letters denote significant differences (*P* < 0.05, one‐way ANOVA). (G) GUS staining of WT and Pro*FtCES18*::GUS transgenic Arabidopsis seedlings.

A total of 30 annotated genes are located within this defined LD region (Table S1). Functional annotation and classification of these genes based on Gene Ontology (GO) terms indicated that the majority are involved in cellular and metabolic processes (Figure S1a). KEGG pathway classification showed that these candidate genes include representatives annotated to “plant–pathogen interaction” and “plant hormone signal transduction” pathways (Figure S1b), broadly participating in plant biotic and abiotic stress responses (Mutuku & Nose, 2012; Rojas et al., 2014). Furthermore, functional annotations revealed that some genes within this region have been reported to participate in plant disease resistance in other species, such as the AP2‐like ethylene‐responsive transcription factor *FtAP2* (*FtPinG0809054800.01*, (Xu et al., 2011)) and CXE carboxylesterase *FtCXE* (*FtPinG0809058800.01* (Ko et al., 2016)). Interestingly, temporal transcriptome analysis of Tartary buckwheat infected with *R. solani* showed that the expression levels of most genes in this region gradually decreased over the interaction time. However, the expression of four specific genes (*FtPinG0809058500.01*, *FtPinG0809059200.01*, *FtPinG0809059600.01*, and *FtPinG0809060200.01*) was significantly upregulated as the infection progressed (Figure 1C, Figure S2, and Table S2). To rule out potential circadian clock‐driven background fluctuations and confirm whether this dynamic upregulation was specifically triggered by fungal invasion, we conducted time‐course RT‐qPCR analysis under both *R. solani*‐challenged and time‐matched mock‐inoculated conditions across 0, 6, 14, and 22 h post‐inoculation (hpi). In mock‐treated leaves, the transcript abundances of all four candidate genes remained low and remarkably stable throughout the time course. In contrast, *R. solani* challenge triggered a rapid induction of these genes, with their transcript levels increasing significantly compared to their time‐matched mock controls (Figure S3). These results demonstrated that these four candidate genes are specifically and strongly responsive to *R. solani* attack and likely play active roles in Tartary buckwheat defense.

Given the crucial role of plant hormones, especially JA, in plant defense mechanisms (Cox, 2022; Ma et al., 2022; Zhang et al., 2021; Zhang et al., 2024; Q. Zhao, Liu, et al., 2023), we further investigated the expression patterns of these four genes in a JA‐treated transcriptome dataset to narrow down the candidates. The results showed that among the four genes, only *FtPinG0809058500.01* exhibited a gradual increase in expression with prolonged JA treatment (Figure 1D, Table S3), implying that this gene might mediate Tartary buckwheat disease resistance via the JA signaling pathway. Annotation against the NR database indicated that this gene encodes a carboxylesterase (CES18). CESs are a class of hydrolases with an α/β‐fold domain that catalyze the hydrolysis of esters and amides (Figure 1E), playing vital roles in regulating herbicide bioactivation, stem branching, detoxification, and pathogen defense (Hatfield et al., 2016; Seo et al., 2018; Wang et al., 2017; Xu et al., 2021). Tissue‐specific expression analysis revealed that *FtCES18* was expressed across various plant tissues but exhibited a distinct tissue preference. Notably, its expression level was highest in the stem, followed by the roots (Figure 1F). The GUS staining results (Figure 1G) were highly consistent with the quantitative data, with intense dark blue GUS signals strongly localized in the root network and the hypocotyl/stem base regions of the plants. Considering that *R. solani* is a destructive soil‐borne pathogen that primarily attacks the roots and stem bases of plants, this strongly implies that *FtCES18* plays an important defensive role against the invasion of this pathogen. Collectively, we ultimately identified *FtPinG0809058500.01* (*FtCES18*) as the core candidate gene for further investigation of its function and molecular mechanisms in Tartary buckwheat disease resistance.

### *FtCES18* enhances *R. solani* resistance by remodeling ROS homeostasis

To directly evaluate the disease resistance capability of *FtCES18*, we generated three independent *FtCES18*‐overexpressing *Arabidopsis* lines (OE1, OE2, OE3). RT‐qPCR analysis confirmed the marked overexpression of *FtCES18* in the transgenic lines, displaying an approximate 60‐ to 105‐fold increase in transcript abundance across three independent lines (OE1, OE2, and OE3) compared to the WT control (Figure S4a). Under non‐stress growth conditions, these *FtCES18‐*OE plants exhibited normal vegetative growth, development, and leaf morphology, showing no discernible phenotypic defects or developmental delay relative to WT plants. We conducted detached‐leaf inoculation experiments using *R. solani*. At 48 h post‐inoculation (hpi), wild‐type (WT) leaves developed severe, rapidly expanding necrotic lesions around the inoculation sites. In stark contrast, the disease symptoms in the OE lines were significantly restricted, resulting in lesion areas that were markedly smaller than those of the WT plants (Figure 2A, upper panel; Figure 2B).

**Figure 2:**
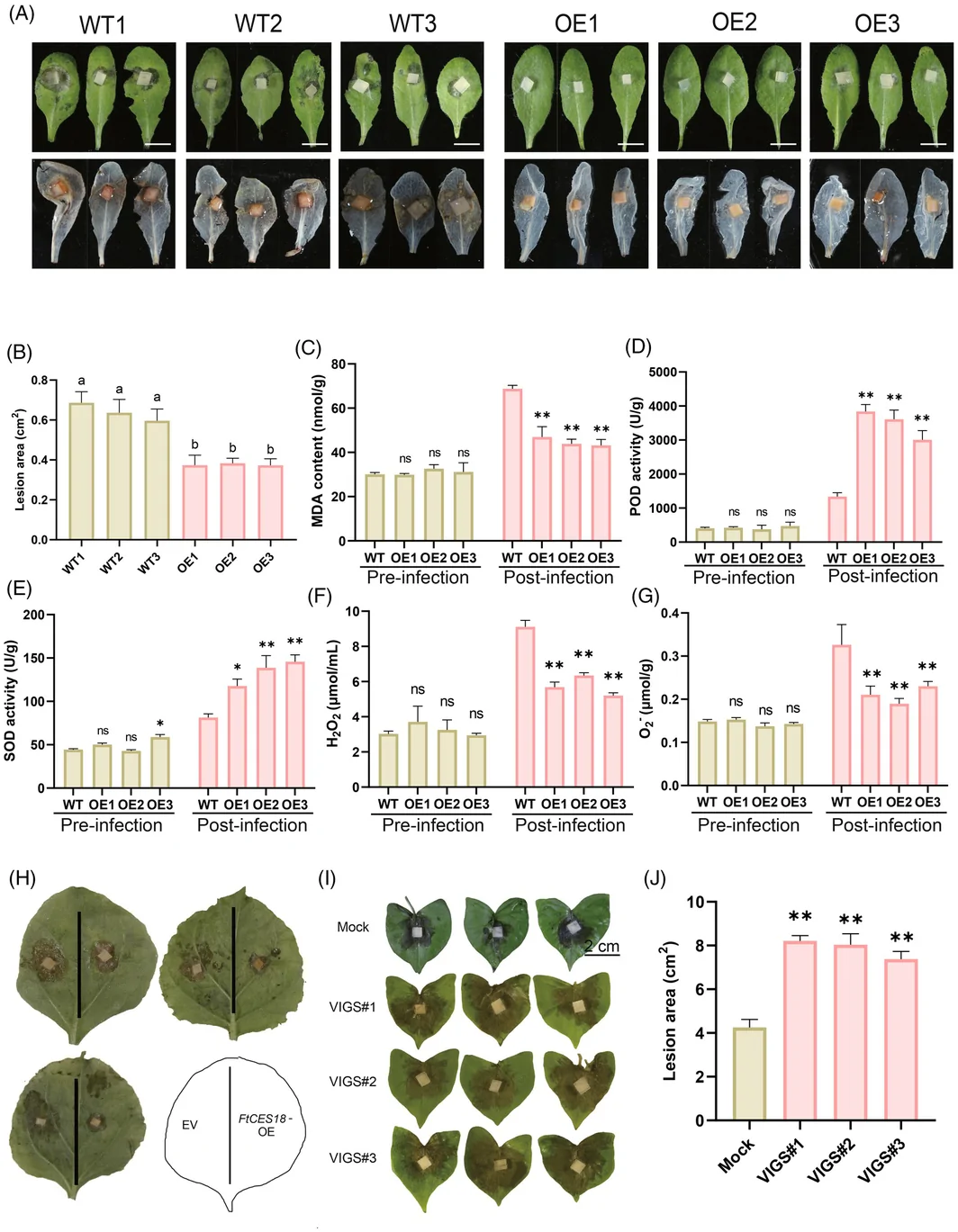
Disease resistance evaluation and antioxidant enzyme activities of *FtCES18* transgenic plants. (A, B) Disease symptoms (A) and lesion area quantification (B) on detached leaves of WT and *FtCES18*‐overexpressing (OE1–3) Arabidopsis lines inoculated with *R. solani*. Different lower‐case letters indicate significant differences (*P* < 0.05, one‐way ANOVA), the length of the scale bar is 1 cm. (C–G) MDA content (C), POD activity (D), SOD activity (E), H_2_O_2_ content (F), and O_2_‐ content (G) in WT and OE Arabidopsis leaves before (Pre‐infection) and after (Post‐infection) pathogen inoculation. (H) Transient expression assay in *N. benthamiana* leaves. The left and right halves were infiltrated with *Agrobacterium* carrying the empty vector (EV) and *FtCES18* overexpression construct (*FtCES18*‐OE), respectively, followed by pathogen inoculation. (I, J) Disease symptoms (I) and lesion area quantification (J) on detached leaves of Mock and *FtCES18*‐silenced (VIGS#1–3) Tartary buckwheat. Data in bar graphs are presented as mean ± SD. Asterisks denote significant differences compared with the WT or Mock (**P < 0.01; *P < 0.05; ns, not significant; Student's t‐test).

As a typical necrotrophic pathogen, *R. solani* induces marked accumulation of reactive oxygen species (ROS) during early infection to induce host cell necrosis for nutrient acquisition (Mengiste, 2012; Taheri & Tarighi, 2011). To investigate whether *FtCES18* mitigates pathogen‐induced oxidative stress, we performed in situ 3,3′‐diaminobenzidine (DAB) histochemical staining to detect hydrogen peroxide (H_2_O_2_) accumulation (Daudi & O'Brien, 2012; Vanacker et al., 2000). The results demonstrated that the infected WT leaves accumulated extensive dark brown precipitates in and around the lesions, reflecting pronounced H_2_O_2_ accumulation. Conversely, the DAB staining signals were substantially diminished in the OE leaves and largely confined to the immediate vicinity of the inoculation sites (Figure 2A, lower panel). In line with these histological observations, physiological and biochemical assays revealed that prior to infection (pre‐infection), there were no significant differences in malondialdehyde (MDA) content or the activities of peroxidase (POD) and superoxide dismutase (SOD) between the WT and OE lines (Figure 2C–E, Table S4). However, following pathogen challenge (Post‐infection), the WT plants exhibited a sharp increase in MDA accumulation—a hallmark of cell membrane lipid peroxidation damage—whereas the OE lines successfully maintained significantly lower MDA levels (Figure 2C). Concurrently, activities of antioxidant enzymes (POD and SOD) were significantly induced in the OE lines post‐infection, reaching higher levels than those in the WT plants (Figure 2D,E). Consistent with this enhanced antioxidant system, pathogen infection triggered a severe oxidative burst in WT leaves, whereas the accumulation of both H_2_O_2_ and O_2_•^−^ was markedly suppressed in the *FtCES18*‐OE lines, remaining at significantly lower levels compared to the WT (Figure 2F,G). These data demonstrate that overexpressing *FtCES18* empowers the host's antioxidant defense machinery, enabling the efficient scavenging of pathogen‐induced excessive ROS, thereby protecting cells from irreversible oxidative damage and necrosis.

To further corroborate the broad‐spectrum disease resistance efficacy of this gene across diverse plant systems, we conducted a transient overexpression assay in *N. benthamiana* leaves. Following *R. solani* inoculation, the leaf halves infiltrated with the *FtCES18* overexpression construct (*FtCES18*‐OE) developed significantly smaller lesions compared to the halves infiltrated with the empty vector (EV) (Figure 2H). More importantly, to perform reverse genetic validation within the native Tartary buckwheat system, we employed virus‐induced gene silencing (VIGS) to knock down *FtCES18* expression (Figure S4b). Phenotypic evaluations revealed that, compared with the mock control, all three independent silenced leaves (VIGS#1, VIGS#2, and VIGS#3) exhibited a highly susceptible phenotype upon *R. solani* inoculation, characterized by rapid leaf collapse and the formation of prominent necrotic lesions (Figure 2I,J). Taken together, these comprehensive gain‐ and loss‐of‐function analyses demonstrate that *FtCES18* acts as a positive regulator mediating plant resistance against *R. solani* infection.

### FtCES18 mediates MeJA hydrolysis to enhance disease resistance in Tartary buckwheat

To investigate the biological function and underlying mechanism of *FtCES18*, we first determined its subcellular localization. Confocal imaging demonstrated that the GFP‐fused FtCES18 protein specifically localized to the endoplasmic reticulum (ER) in *N. benthamiana* cells, co‐localizing with an ER marker (Figure 3A). To further explore the evolutionary characteristics of the candidate gene *FtCES18*, a phylogenetic tree was constructed using CES18 protein sequences from Tartary buckwheat and various other plant species (Figure 3B). Phylogenetic analysis revealed that FtCES18 clustered predominantly with CES18 homologs from other dicotyledonous plants. Notably, FtCES18 exhibited the highest evolutionary proximity to the CES18 protein from *Rumex salicifolius*. This high degree of phylogenetic consistency and sequence conservation suggests that FtCES18 likely shares similar biological functions with its orthologs in closely related species, playing a highly conserved role in plant adaptive evolution and defense mechanisms.

**Figure 3:**
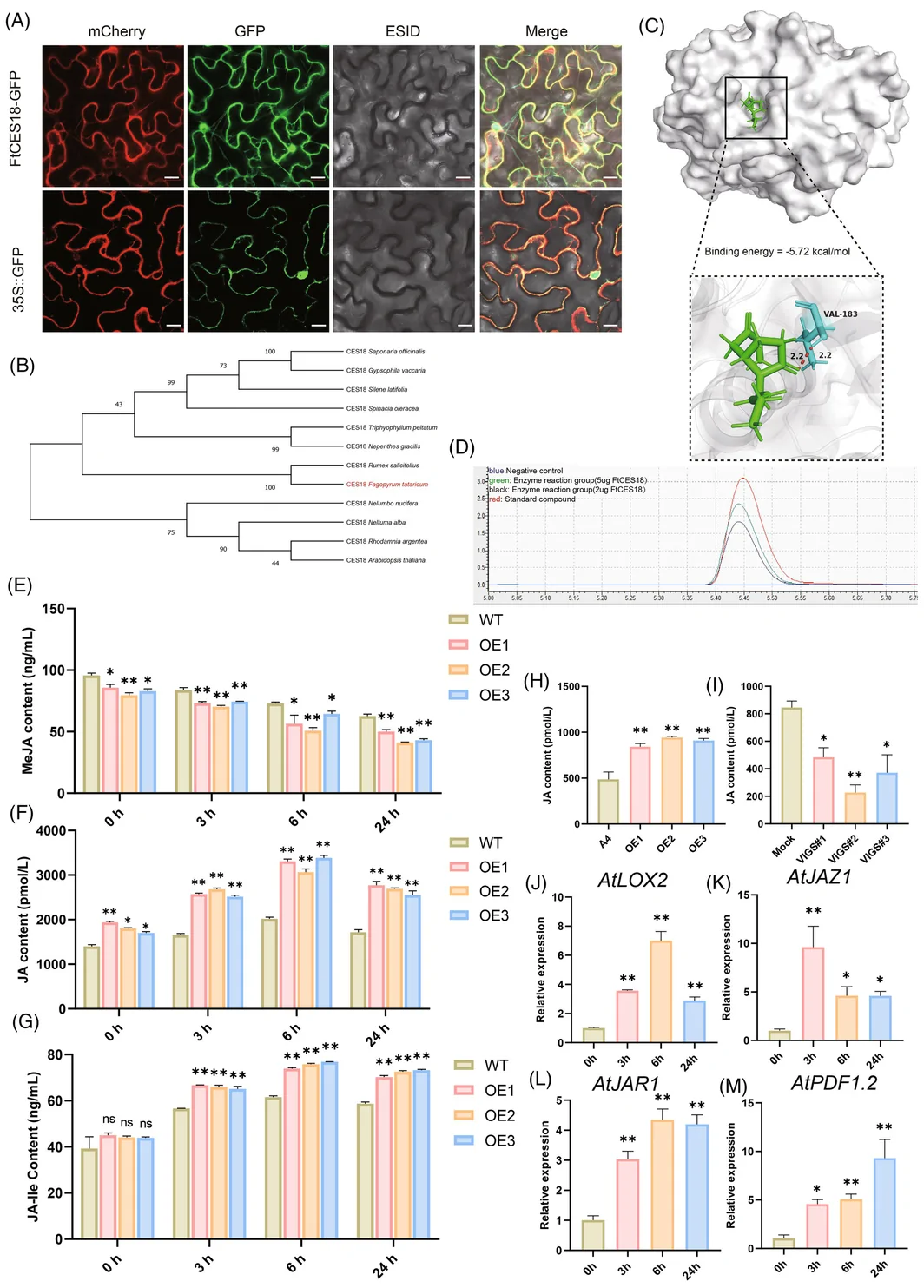
*FtCES18* regulates jasmonate metabolism and orchestrates downstream defense signaling. (A) Subcellular localization of *FtCES18* in *N. benthamiana* leaf epidermal cells. *FtCES18*‐GFP was co‐expressed with an endoplasmic reticulum (ER) marker (mCherry); scale bar = 20 μm. (B) Phylogenetic tree of *FtCES18* and homologous carboxylesterases from other plant species. (C) Molecular docking simulation of *FtCES18* with methyl jasmonate (MeJA), showing a predicted binding energy of −5.72 kcal/mol. (D) In vitro enzymatic activity assay of recombinant *FtCES18* confirmed by LC–MS/MS (m/z 209.20 > 109.00, ESI‐, CE = 19.0 eV). Representative multiple reaction monitoring (MRM) chromatograms of the reaction mixtures. The chromatographic traces represent the authentic jasmonic acid (JA) standard (red), the enzymatic reaction products generated by two distinct protein concentrations (5.0 μg [green] and 2.0 μg [black] GST‐*FtCES18*), and the negative control incubated with purified GST protein alone (blue). (E–G) Dynamic accumulation of jasmonate metabolites—including (E) MeJA, (F) free JA, and (G) JA‐Ile—in leaves of WT and *FtCES18*‐overexpressing (OE) Arabidopsis lines at 0, 3, 6, and 24 h post‐inoculation (hpi) with *R. solani*. (H) Endogenous JA content in wild‐type and *FtCES18*‐overexpressing Tartary buckwheat hairy root lines. (I) Endogenous JA content in control (Mock/TRV) and *FtCES18*‐silenced (VIGS) Tartary buckwheat leaves. (J–M) Relative transcript abundance of JA‐responsive genes including (J) *AtLOX2*, (K) *AtJAZ1*, and (L) *AtJAR1*, as well as a defense marker gene (M) *AtPDF1.2*, in *Arabidopsis* leaves at 0, 3, 6, and 24 hpi with *R. solani*. Data in bar graphs are presented as mean ± SD. Asterisks denote significant differences compared with the respective controls (*P < 0.05, **P < 0.01; Student's t‐test).

It is well‐documented that one of the primary biochemical functions of carboxylesterases (CES) is to catalyze the hydrolysis of MeJA into JA (Cao et al., 2019; Chaffin et al., 2024; Koo & Howe, 2009; Zhang et al., 2023). To validate the interaction potential between FtCES18 and MeJA at the structural level, molecular docking analysis was performed between the three‐dimensional model of the FtCES18 protein and the MeJA molecule (Figure 3C). The docking simulation demonstrated that MeJA was well‐accommodated within the active binding pocket on the surface of the FtCES18 protein. The calculated binding energy was −5.72 kcal/mol, indicating a strong spontaneous binding affinity between the enzyme and the substrate. A magnified view of the binding interface further revealed the crucial interacting amino acid residues: the MeJA molecule forms a tight intermolecular interaction (with a distance of 2.2 Å) with the valine residue (VAL‐183) of FtCES18. These structural biology findings confirm that FtCES18 possesses the requisite spatial conformation to bind and catalyze MeJA, providing vital theoretical support for its role as a hydrolase in regulating *in vivo* JA homeostasis. To determine whether FtCES18 functions as a bona fide jasmonate methylesterase, we heterologously expressed GST‐tagged FtCES18 recombinant protein in *E. coli* (BL21) and performed *in vitro* enzymatic activity assays using MeJA as the substrate (Figure S5). The reaction products were qualitatively analyzed via LC–MS/MS under multiple reaction monitoring (MRM) mode. As shown in Figure 3D, incubation of purified GST‐FtCES18 fusion protein with MeJA led to the formation of free JA, displaying a strong chromatographic peak in the qualifier transition (*m/z*: 209.20 > 109.00). Both the 2‐ and 5‐μg GST‐FtCES18 reactions produced a JA‐associated chromatographic peak that co‐eluted with the authentic JA standard at approximately 5.45 min (ΔRT < 0.01 min), whereas no corresponding peak was detected in the GST control. To further confirm the chemical identity of the reaction product, MS/MS product ion scanning was performed on the precursor ion at *m/z* 209.20 ([M‐H]^−^ of free JA) at a collision energy of 25.0 eV. As shown in Figure S6, collision‐induced dissociation of *m/z* 209.15 yielded two predominant characteristic fragment ions at *m/z* 59.10 (base peak) and *m/z* 109.00, which was consistent with the secondary mass spectral fragmentation pattern of authentic JA. These results demonstrate that FtCES18 possesses the catalytic capacity to efficiently convert MeJA into free JA.

To further verify *in vivo* whether FtCES18 functions to promote JA accumulation, we generated *FtCES18*‐overexpressing (OE) *A. thaliana* and Tartary buckwheat hairy root lines (Figures S4a and S7). Targeted profiling of jasmonates demonstrated that overexpressing *FtCES18* significantly accelerated MeJA consumption while triggering a robust accumulation of downstream jasmonate species upon *R. solani* infection (Figure S8, Table S5, Figure 3E–G). Specifically, the substrate MeJA levels were consistently lower in the three OE lines compared to the WT across all time points (0, 3, 6, and 24 h post‐inoculation, hpi), decreasing progressively with pathogen challenge (Figure 3E). Conversely, free JA contents were significantly elevated in the OE lines relative to WT at all time points, exhibiting a rapid accumulation that peaked at 6 hpi (3005–3452 pmol/L in OE lines vs. 1986–2056 pmol/L in WT) before slightly declining at 24 hpi (Figure 3F). Concurrently, the bioactive ligand JA‐Ile displayed a synchronized accumulation pattern; while no significant difference was observed under mock conditions (0 hpi), JA‐Ile levels surged rapidly post‐inoculation and reached markedly higher levels in OE lines than in WT at 3, 6, and 24 hpi (peaking at 73.6–76.8 ng/mL in OE vs. 61.1–62.0 ng/mL in WT at 6 hpi) (Figure 3G). This indicates that *FtCES18* overexpression not only elevates basal JA levels but also sensitizes the plants, leading to a more pronounced JA response upon pathogen infection. Similarly, the JA content in *FtCES18*‐overexpressing Tartary buckwheat hairy root lines was significantly higher than that in WT (Figure 3H, Table S6); conversely, in Tartary buckwheat lines where *FtCES18* was silenced via VIGS, the JA content exhibited a significant decrease (Table S7, Figure 3I). Consistently, profiling of additional jasmonate species in these materials showed that overexpressing *FtCES18* significantly reduced substrate MeJA levels while boosting bioactive JA‐Ile accumulation in hairy roots (Figure S9a,b). Conversely, *FtCES18* silencing led to marked MeJA accumulation alongside a significant reduction in JA‐Ile contents in leaves (Figure S9c,d). Taken together, these complementary gain‐ and loss‐of‐function analyses demonstrate that *FtCES18* accelerates MeJA hydrolysis to drive downstream JA and JA‐Ile accumulation in *planta*.

To further validate the regulatory cascade of *FtCES18* on JA metabolism and downstream signaling pathways at the molecular level, we examined the expression levels of key genes involved in JA metabolism and signaling (*AtLOX2*, *AtJAZ1*, *AtJAR1*) (Figure S10) (Chauvin et al., 2013; Liu et al., 2021; Meesters et al., 2014; Staswick et al., 2002; Wasternack & Feussner, 2018; Wu et al., 2024), as well as the downstream classic defense marker gene (*AtPDF1.2*) in *Arabidopsis* using RT‐qPCR (Ndamukong et al., 2007; Singh & Nandi, 2022; Wang et al., 2015). To further elucidate the temporal dynamics of the JA signaling cascade following pathogen infection, we examined the expression dynamics of genes involved in JA biosynthesis, signal transduction, and defense responses in *FtCES18*‐overexpressing *Arabidopsis* inoculated with *R. solani* at 0, 3, 6, and 24 h post‐inoculation (hpi) (Figure 3J–M). Upon fungal challenge, the transcript levels of *AtLOX2* and *AtJAR1* were rapidly induced, both reaching peak expression levels at 6 hpi (approximately 6.9‐fold and 4.3‐fold upregulation, respectively) (Figure 3J,L), which tightly aligned with the accumulation dynamics of free JA and JA‐Ile. Notably, the transcriptional repressor gene *AtJAZ1* exhibited an immediate and substantial burst as early as 3 hpi (~9.5‐fold induction) before maintaining an elevated level at 6 and 24 hpi (Figure 3K), reflecting a rapid primary negative feedback mechanism. Conversely, the downstream defense‐executing gene *AtPDF1.2* displayed a progressive and sustained accumulation over time, reaching its maximum expression at 24 hpi (~9.2‐fold increase) (Figure 3M). Together, these temporal expression patterns demonstrate that *R. solani* infection triggers a highly synchronized transcriptional cascade of the JA pathway, advancing from rapid early signal activation to robust late‐stage antimicrobial defense expression.

### FtCES18‐mediated JA signaling reprograms flavonoid metabolism in Tartary buckwheat

Our previous results established that *FtCES18* triggers an endogenous JA burst in Tartary buckwheat via MeJA hydrolysis to scavenge excessive ROS. Meanwhile, flavonoids are universally recognized as the most critical non‐enzymatic antioxidants *in planta*. To investigate whether JA signaling directly drives flavonoid biosynthesis in Tartary buckwheat, we first measured the total flavonoid content in leaves treated with exogenous MeJA. The results demonstrated that MeJA treatment significantly induced a substantial accumulation of total flavonoids compared with the mock control (Figure S11, Figure 4A), confirming that flavonoid metabolism is a core secondary metabolic event responding to JA signaling in this species. To elucidate its transcriptional regulatory basis, we analyzed the expression patterns of flavonoid biosynthetic structural genes using a JA‐treated temporal transcriptome dataset. The heatmap (Figure 4B, Table S8) clearly displays that with prolonged exogenous JA treatment (1 h, 4 h, and 12 h), nearly all key structural genes across the flavonoid biosynthesis pathway were substantially and coordinately activated. This orchestrated upregulation encompasses not only upstream genes responsible for the phenylpropanoid skeleton (*PAL*, *C4H*, and *4CL*) but also core skeleton and downstream modification genes (*CHS*, *CHI*, *F3H*, and *FLS*), as well as terminal glycosyltransferases (e.g., *F3GT*, *F3RT*, and *RT*) (Shen et al., 2022; Treutter, 2005). This indicates that JA signaling broadly activates flavonoid biosynthetic machinery at the transcriptional level.

**Figure 4:**
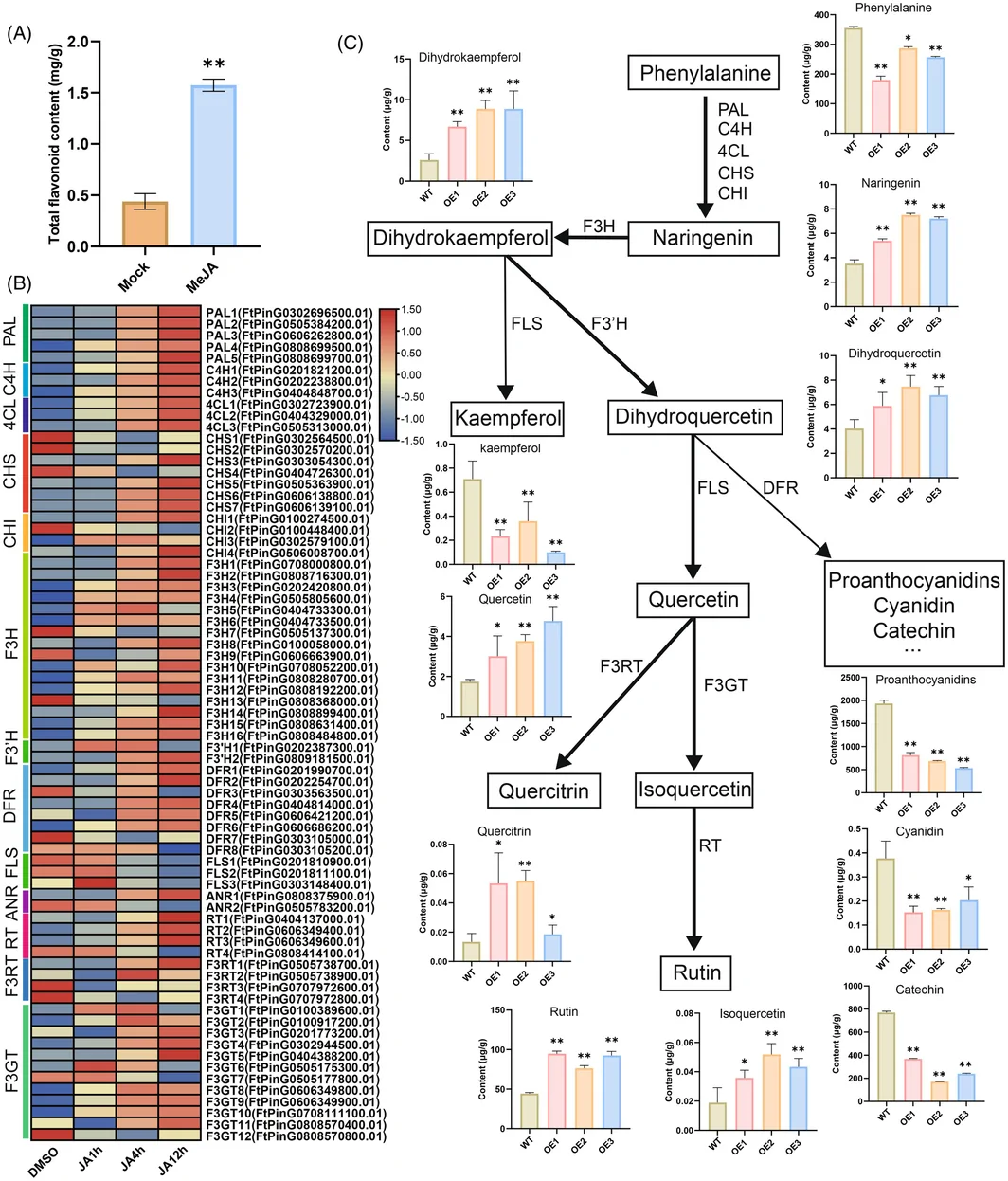
FtCES18‐mediated JA signaling drives the reprogramming of flavonoid metabolic flux. (A) Total flavonoid content in Tartary buckwheat leaves treated with 100 μM MeJA. (B) Expression heatmap of key structural genes in the flavonoid biosynthesis pathway under exogenous JA treatment at 0 (DMSO), 1, 4, and 12 h. *PAL*, phenylalanine ammonia‐lyase; *C4H*, cinnamate 4‐hydroxylase; *4CL*, 4‐coumarate:CoA ligase; *CHS*, chalcone synthase; *CHI*, chalcone isomerase; *F3H*, flavanone 3‐hydroxylase; *F3'H*, flavonoid 3'‐hydroxylase; *DFR*, dihydroflavonol 4‐reductase; *FLS*, flavonol synthase; *ANR*, anthocyanidin reductase; *F3GT*, flavonol 3‐O‐glucosyltransferase; *F3RT*, flavonol 3‐O‐rhamnosyltransferase; *RT*, rhamnosyltransferase. (C) Schematic representation of the flavonoid metabolic pathway integrated with targeted metabolite quantification in wild‐type and *FtCES18*‐overexpressing Tartary buckwheat hairy root lines. Bar graphs display the relative contents of specific metabolites measured by HPLC. Thick and thin arrows indicate enhanced and reduced metabolic fluxes, respectively. Data in bar graphs are presented as mean ± SD. Asterisks denote significant differences compared with the respective controls (*P < 0.05, **P < 0.01; Student's t‐test).

To further precisely delineate the substantive impact of the FtCES18‐mediated endogenous JA burst on the metabolic pathway, we performed targeted HPLC to quantify key flavonoid intermediates and terminal products in wild‐type (WT) *FtCES18*‐overexpressing Tartary buckwheat hairy root lines (OE1‐OE3) (Figure 4C, Table S9). The mapped metabolic pathway revealed a classic pattern of “metabolism redirection”: in the overexpression lines, the content of the free precursor amino acid for the flavonoid biosynthesis pathway, phenylalanine, exhibited a highly significant decrease. This reduction in the upstream substrate is likely attributed to the overexpression of downstream biosynthetic enzymes (e.g., *PAL*, *C4H*, and *CHS*), which leads to rapid substrate depletion. Subsequently, core midstream products, such as naringenin, dihydrokaempferol, and dihydroquercetin, all exhibited highly significant increases in the OE lines.

Interestingly, the metabolism exhibited highly specific channeling at downstream branch points. The contents of side‐branch products, such as proanthocyanidins, catechin, and cyanidin, were significantly suppressed, which tightly correlates with the attenuation of DFR transcriptional levels in the OE lines. Simultaneously, kaempferol accumulation was also significantly restricted. Conversely, the bulk of the metabolism was channeled into the main pathway of “quercetin and its glycosylated derivatives,” leading to a marked accumulation of defensive flavonoids (quercetin, quercitrin, isoquercetin, and rutin) in the overexpression lines (indicated by thick arrows in Figure 4C). Taken together, these results indicate that the FtCES18‐mediated JA signaling does not indiscriminately elevate all secondary metabolites, but rather precisely channels the flavonoid metabolism of Tartary buckwheat predominantly toward the downstream rutin/quercetin defensive branch, which possesses potent antioxidant and disease‐resistance activities.

### The transcription factor FtMYB32 directly binds to and positively regulates *FtCES18*

Since the expression of *FtCES18* was induced upon *R. solani* infection (Figure S2), we performed temporal co‐expression clustering analysis on the Tartary buckwheat–*R. solani* interaction transcriptome to systematically screen for upstream transcription factors that regulate *FtCES18*. KEGG pathway enrichment analysis of the *FtCES18*‐containing Cluster 8 module (Figure 5A, Table S10) revealed that genes within this co‐expression network were significantly enriched in immunity‐related pathways, such as “Protein processing in endoplasmic reticulum” (Korner et al., 2015; Y. Yang, Zhao, et al., 2025) and “Endocytosis” (Duan et al., 2022; Gu et al., 2017; Leborgne‐Castel et al., 2010) (Figure 5B). Furthermore, GO classification demonstrated that these co‐expressed genes are broadly involved in core biological processes, including “metabolic process” (Leonetti & Molinari, 2020) and “response to stimulus” (Zhou & Zhang, 2020) (Figure S12). These pathways serve as hubs in plant‐pathogen perception (e.g., vesicular trafficking of transmembrane receptors) and the proper folding and secretion of defense‐related proteins. This strongly implies that this module harbors core transcription factors that directly respond to pathogen invasion and orchestrate downstream disease resistance networks.

**Figure 5:**
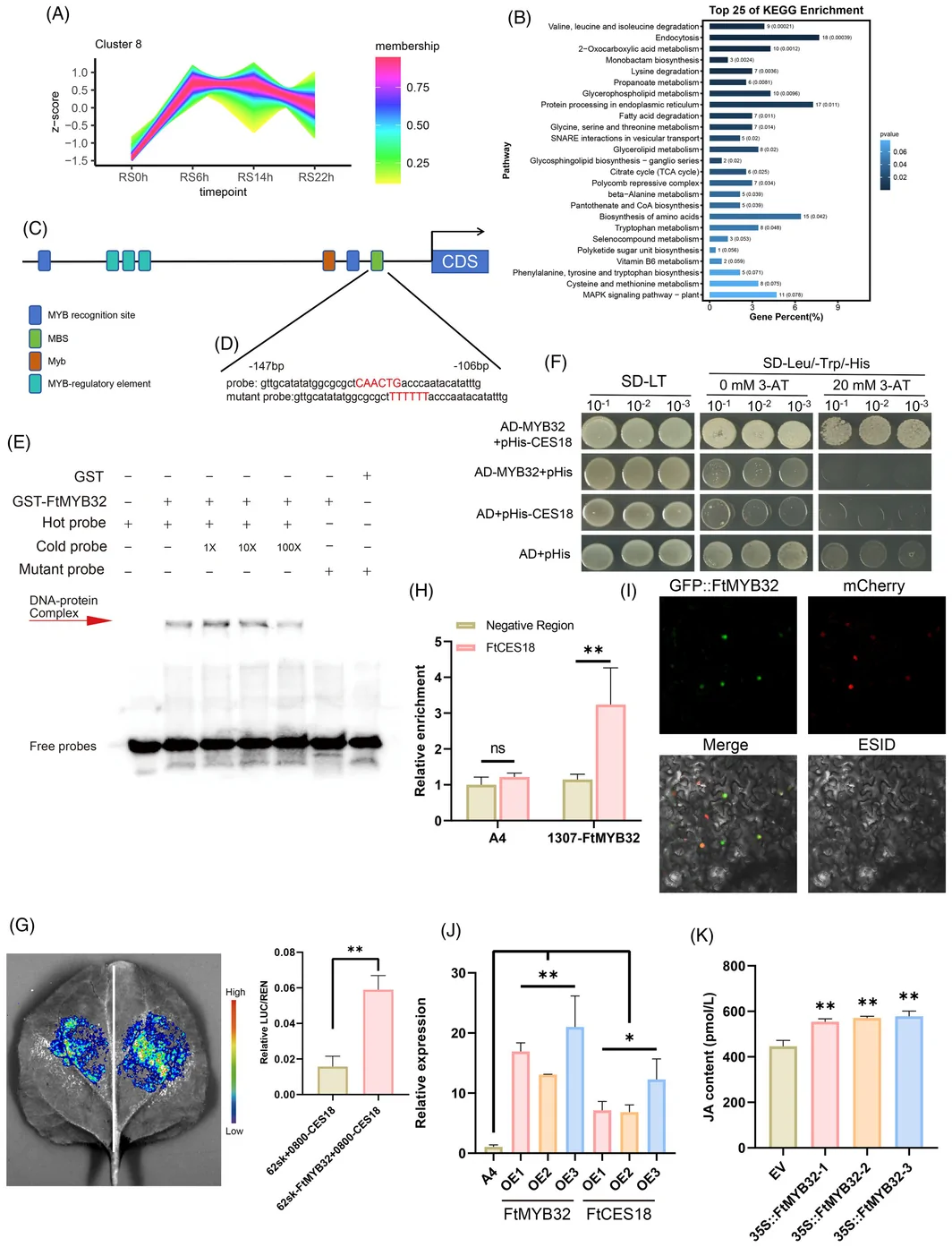
Characterization of the transcription factor FtMYB32 and its interaction with the *FtCES18* promoter. (A, B) Expression pattern (A) and KEGG pathway enrichment (B) of the co‐expressed gene cluster (Cluster 8) during *R. solani* infection. (C, D) Schematic diagram of MYB‐associated cis‐elements in the *FtCES18* promoter (C) and sequences of the wild‐type and mutant probes used for EMSA (D). The MBS core element is highlighted in red. (E) EMSA demonstrating the direct binding of the GST‐FtMYB32 recombinant protein to the MBS element. (F) Yeast one‐hybrid (Y1H) assay verifying the binding of FtMYB32 to the *FtCES18* promoter. Transformed yeast cells were grown on SD‐Leu/−Trp/−His medium supplemented with 0 or 20 mM 3‐AT. (G) Dual‐luciferase reporter assay showing the transcriptional activation of the *FtCES18* promoter by FtMYB32 in *N. benthamiana* leaves. (H) ChIP‐qPCR assay demonstrating the in vivo binding of FtMYB32 to the *FtCES18* promoter in 1307‐FtMYB32 hairy roots compared to the A4 control and a non‐binding negative region. (I) Subcellular localization shows that the GFP::FtMYB32 fusion protein is exclusively localized to the nucleus. (J) Transcript abundance of FtMYB32 and downstream *FtCES18* in control (A4) and three independent *FtMYB32*‐overexpressing (*FtMYB32*‐OE) Tartary buckwheat hairy root lines. (K) Transient overexpression of FtMYB32 significantly increases endogenous JA content compared to the mock control. Data are presented as mean ± SD. Asterisks denote significant differences (**P < 0.01; ns, not significant; Student's t‐test).

Accordingly, we conducted in *silico* prediction of *cis*‐acting elements within the 2000‐bp promoter region of *FtCES18*. The results revealed multiple tandem MYB‐specific binding elements (MBS elements) within the promoter sequence (Figure 5C, Table S11). Given that the MYB transcription factor family is highly conserved across plant species and is widely implicated in transcriptional reprogramming during both biotic and abiotic stress responses (Liu et al., 2013; Mengiste et al., 2003), we targeted MYB transcription factors within Cluster 8 that exhibited a highly synchronized co‐expression pattern (Pearson's *r* > 0.8) with *FtCES18*. This rigorous screening ultimately led to the identification and cloning of *FtMYB32*.

To verify whether FtMYB32 directly regulates *FtCES18* at the molecular level, we conducted a series of *in vitro* and *in vivo* protein–DNA interaction assays. Initially, biotin‐labeled oligonucleotide probes containing the core MBS elements from the *FtCES18* promoter were synthesized for an electrophoretic mobility shift assay (EMSA) (Figure 5D). The *in vitro* binding results clearly demonstrated that the purified recombinant FtMYB32 protein could specifically bind to the MBS‐containing probes, resulting in a distinct shifted band (Figure 5E). Subsequently, we performed *in vivo* validations using yeast one‐hybrid (Y1H) and dual‐luciferase (Dual‐LUC) reporter systems. The Y1H assay indicated that the FtMYB32 protein fused with an activation domain could directly bind to the *FtCES18* promoter sequence and activate the expression of downstream reporter genes (Figure 5F). In the dual‐luciferase assay, co‐expression of the *FtMYB32* effector construct (62SK‐FtMYB32 + 0800‐*FtCES18*) in tobacco leaves significantly upregulated the relative LUC/REN luciferase activity compared with the empty vector control (62SK + 0800‐*FtCES18*) (Figure 5G). To further investigate whether FtMYB32 directly binds to the *FtCES18* promoter *in vivo*, chromatin immunoprecipitation followed by quantitative PCR (ChIP‐qPCR) assays were conducted using the 1307‐*FtMYB32* hairy root lines, with A4 hairy roots serving as the negative background control. As shown in Figure 5H, the *FtCES18* promoter region harboring the MBS element exhibited a significant enrichment (more than threefold increase) in the 1307‐*FtMYB32* lines compared to the non‐binding negative control region (**P < 0.01). In contrast, no statistically significant difference in relative enrichment was observed between the *FtCES18* promoter region and the negative control region in A4 control lines (Figure 5H). These ChIP‐qPCR findings provide compelling *in vivo* evidence demonstrating that FtMYB32 directly targets and associates with the promoter of *FtCES18*. Consistent with its function as a transcriptional regulator, FtMYB32 was observed to localize strictly within the plant cell nucleus (Figure 5I).

Subsequently, we further characterized the biological function of *FtMYB32*. Intriguingly, by re‐analyzing the GWAS data for metabolic traits, we identified a significant association signal for quercetin content located adjacent to the *FtMYB32* genomic locus (Figure S13). To further validate this transcriptional regulation in *vivo* within the homologous Tartary buckwheat system, we analyzed the expression levels of both *FtMYB32* and *FtCES18* in transgenic Tartary buckwheat hairy root lines overexpressing *FtMYB32* (*FtMYB32*‐OE). The result demonstrated that the transcript levels of *FtMYB32* were dramatically elevated by approximately 13‐ to 21‐fold in three independent transgenic lines (*FtMYB32*‐OE1 to *FtMYB32*‐OE3) compared to the wild‐type control hairy roots (A4) (Figure 5J). Concurrently, the expression of the downstream target gene FtCES18 was also significantly upregulated by 7‐ to 12‐fold in these same overexpression backgrounds (Figure 5J). Conversely, when FtMYB32 was silenced via VIGS in Tartary buckwheat leaves (*FtMYB32*‐VIGS), the transcript levels of *FtMYB32* and *FtCES18* were coordinately and significantly downregulated by approximately 50–65% and 30–60%, respectively, compared to the TRV control (Figure S14). Collectively, these complementary *in vivo* gain‐ and loss‐of‐function lines within the homologous system demonstrate that *FtMYB32* directly and positively orchestrates the transcription of *FtCES18*. In addition to promoting *FtCES18* transcription, we investigated whether *FtMYB32* coordinately regulates the downstream metabolic machinery of defensive flavonoids. We quantified the transcript levels of several key genes in the core rutin biosynthetic pathway, including flavanone 3‐hydroxylase (*FtF3H*), flavonoid 3′‐hydroxylase (*FtF3′H*), flavonol synthase (*FtFLS*), and rutin rhamnosyltransferase 1 (*FtRT1*), in the same FtMYB32‐overexpressing hairy root lines (*FtMYB32*‐OE). The result demonstrated that all four pathway‐specific structural genes were significantly and coordinately upregulated in the three independent transgenic lines compared to the wild‐type control (A4) (Figure S15).

To directly evaluate the role of *FtMYB32* in disease resistance, we performed a transient in planta assay using a half‐leaf infiltration method in *N. benthamiana*. Following inoculation with *R. solani*, the leaf sections transiently expressing FtMYB32 exhibited markedly smaller necrotic lesions compared to the control halves infiltrated with the empty vector (Figure S16). Furthermore, subsequent physiological assays demonstrated that transient expression of *FtMYB32* markedly increased endogenous JA levels (Figure 5K). Collectively, these findings demonstrate that FtMYB32 functions as a central hub, orchestrating plant disease resistance by simultaneously modulating JA homeostasis and promoting flavonoid accumulation.

### Natural variation in the *FtMYB32* promoter drives the divergence of disease resistance in Tartary buckwheat

Although the aforementioned functional analyses fully established *FtCES18* as a core effector gene mediating disease resistance in Tartary buckwheat, natural population association analysis revealed a lack of significant single nucleotide polymorphisms (SNPs) associated with the resistance phenotype within the coding and promoter regions of *FtCES18*. This implies that this core gene is highly conserved during evolution (Mukhtar et al., 2011). Therefore, the natural divergence of disease resistance in the Tartary buckwheat population likely originates from genetic variations within its upstream regulatory network (Lu et al., 2026; Xiong et al., 2018). Based on this, we conducted an in‐depth investigation and haplotype analysis of the natural variation in the promoter region of *FtMYB32*, the positive regulator of *FtCES18*. Sequence alignment of the *FtMYB32* promoter across the natural Tartary buckwheat population identified a significant T/C transition SNP upstream of the start codon (at position 45 176 088) (Figure 6A). Based on this SNP, the population could be primarily classified into two distinct haplotypes: hapT (harboring the T allele, TGTAA) and hapC (harboring the C allele, TGCAA). Phenotypic association analysis (Figure 6B) revealed that accessions carrying the homozygous T/T allele (hapT) exhibited a significantly higher disease index against *R. solani* than those with the homozygous C/C allele (hapC), while the heterozygous (T/C) population displayed intermediate resistance. This indicates that hapC is a favorable haplotype for enhancing disease resistance in Tartary buckwheat, and this T/C variation serves as a key genetic basis driving the divergence of disease resistance within the population.

**Figure 6:**
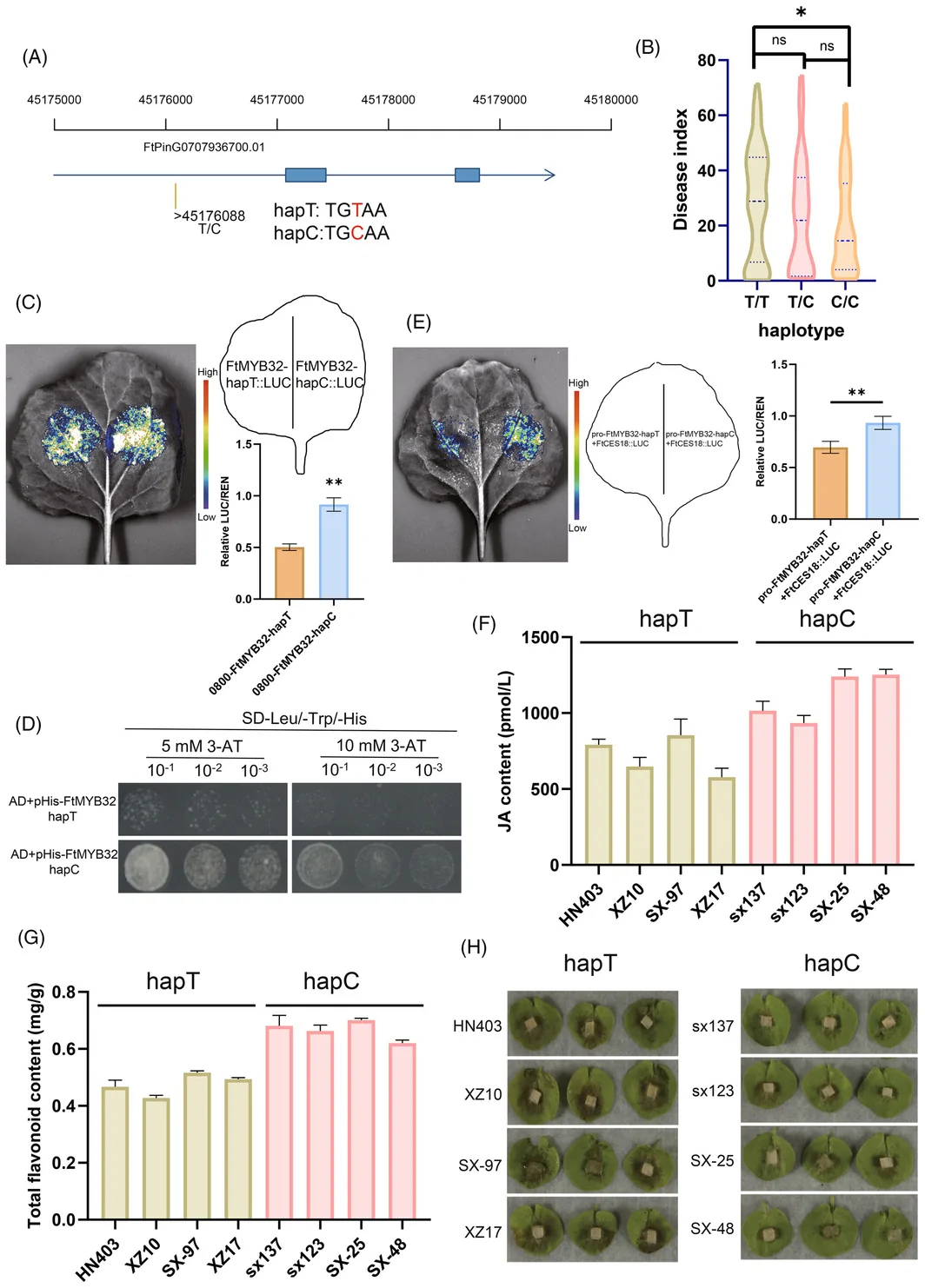
Haplotype analysis and functional evaluation of natural variations in the *FtMYB32* promoter region. (A) Schematic diagram showing the *FtMYB32* gene structure and the T/C single nucleotide polymorphism (SNP) in its promoter region, which defines the two haplotypes (hapT and hapC). (B) Violin plot showing the disease index of Tartary buckwheat accessions carrying different *FtMYB32* genotypes (T/T, T/C, C/C). (C, D) Basal promoter activities of hapT and hapC evaluated by Dual‐LUC reporter assay in *N. benthamiana* leaves (C) and yeast assay (D). (E) Dual‐LUC assay showing the differential activation of the *FtCES18* promoter by *FtMYB32* when driven by its native hapT or hapC promoter. (F, G) Endogenous JA (F) and total flavonoid (G) contents in 8 representative Tartary buckwheat accessions carrying hapT (*n* = 4) or hapC (*n* = 4). (H) Disease symptoms on detached leaves of the representative accessions at 48 h post‐inoculation with *R. solani*. Data in bar graphs are presented as mean ± SD. Asterisks denote significant differences (*P < 0.05, **P < 0.01; ns, not significant). Statistical significance was determined by one‐way ANOVA for (B) and Student's t‐test for others.

To explore the molecular mechanism underlying the phenotypic divergence caused by this SNP, we cloned the *FtMYB32* promoter fragments of both haplotypes and evaluated their transcriptional activities. Both the transient dual‐luciferase (Dual‐LUC) assay in *N. benthamiana* leaves (Figure 6C) and the yeast reporter system (Figure 6D) consistently demonstrated that the promoter activity of *FtMYB32*‐hapC was markedly stronger than that of *FtMYB32*‐hapT. To confirm whether this expression difference induced by promoter variation could be cascaded to the downstream target gene, we co‐expressed the two haplotypes containing their respective native promoters and full‐length coding sequences (pro‐*FtMYB32*‐hapT and pro‐*FtMYB32*‐hapC) as effectors with a reporter gene driven by the *FtCES18* promoter (pro‐*FtCES18*::LUC) *in planta* (Figure 6E). The results indicate that FtMYB32, driven by the endogenous hapC promoter, can significantly enhance the activity of the downstream *FtCES18* promoter.

To further validate this regulatory mechanism and its disease resistance effects in the natural population, we randomly selected four representative accessions from each haplotype group (hapT: HN403, XZ10, SX‐97, XZ17; hapC: sx137, sx123, SX‐25, SX‐48) for multidimensional evaluation. RT‐qPCR analysis revealed that the basal expression levels of *FtMYB32* in the hapC accessions were significantly higher than those in hapT (Figure S17a), which subsequently drove a highly significant upregulation in the expression of its direct target gene, *FtCES18* (Figure S17b). Furthermore, the high expression of *FtCES18* directly promoted a substantial accumulation of JA in the hapC accessions, with levels significantly higher than those in hapT (Figure 6F, Table S12). Additionally, the total flavonoid content in the hapC accessions was also significantly greater than that in the hapT accessions (Figure 6G). Detached leaf inoculation assays (Figure 6H) visually confirmed that the hapC group possessed stronger disease resistance. In summary, the T/C variation in the promoter region directly affects the transcriptional activity of *FtMYB32*; the C allele in hapC significantly enhances the basal expression of this core transcription factor, thereby increasing FtMYB32 protein abundance to activate the downstream disease resistance gene *FtCES18*. This cascade ultimately confers superior disease resistance in Tartary buckwheat, providing an ideal genetic target for future marker‐assisted breeding.

### Natural variation of FtMYB32 in cultivated and wild Tartary buckwheat

To further elucidate the evolutionary trajectory of the *FtMYB32*‐*FtCES18* regulatory module during Tartary buckwheat domestication, we first compared the genomic sequences of *FtCES18* between wild (XZ751, XZ770, XZ631) and cultivated accessions, observing no obvious variation. However, sequence alignment revealed a natural variation in *FtMYB32*, specifically an amino acid deletion in the cultivated accession compared to its wild counterparts (Figure 7A). This truncation originates from a single‐nucleotide deletion in the coding sequence of cultivated *FtMYB32*, which causes a frameshift mutation and introduces a premature stop codon (Figure 7B). Consequently, the cultivated FtMYB32 protein suffers a truncation of 30 amino acids at its highly variable C terminus (Figure 7C). Accordingly, we cloned and compared the *FtMYB32* CDS from the cultivar (PK) and wild accession (XZ751) to investigate their transactivation capacities. Phenotypic and metabolic evaluations demonstrated that *FtCES18* expression was significantly higher in the wild accessions than in the cultivar (Figure 7D), presumably because the full‐length wild‐type FtMYB32 exerts a stronger activation effect on downstream FtCES18. Furthermore, quantification of endogenous JA—the direct enzymatic product of FtCES18—revealed that JA accumulation in the wild accessions was significantly elevated compared to the cultivar (Figure 7E). This JA pool serves as a crucial factor directly contributing to the superior disease resistance of wild Tartary buckwheat.

**Figure 7:**
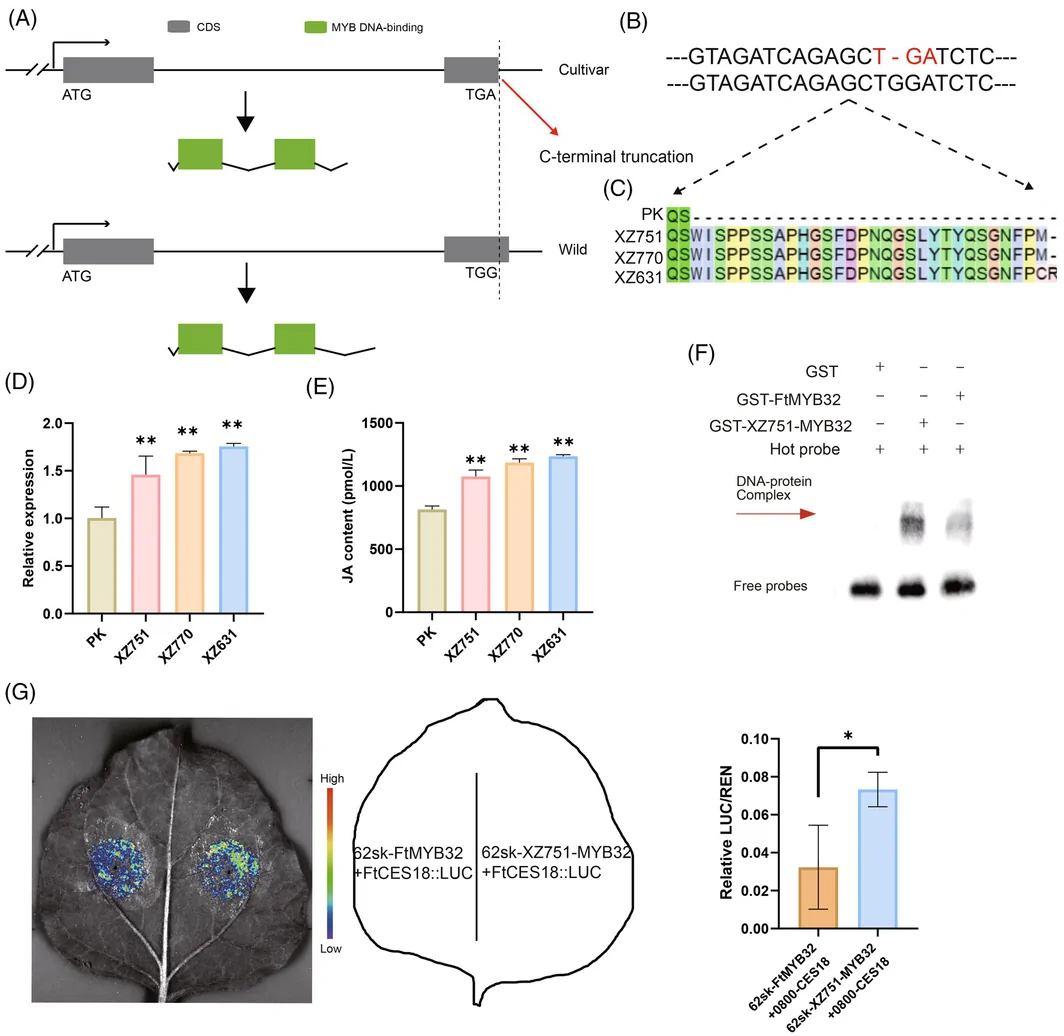
Natural variation of FtMYB32 in cultivated and wild Tartary buckwheat. (A) Schematic diagram of the *FtMYB32* gene structures in cultivated and wild Tartary buckwheat. The red arrow indicates a single‐nucleotide deletion in the cultivar. (B, C) Nucleotide (B) and amino acid (C) sequence alignments of FtMYB32 between the cultivar (PK) and three wild accessions (XZ751, XZ770, and XZ631). (D, E) Relative expression levels of *FtCES18* (D) and endogenous JA contents (E) in the PK cultivar and the three wild accessions. (F) Electrophoretic mobility shift assay (EMSA) of the cultivated (GST‐FtMYB32) and wild (GST‐XZ751‐MYB32) recombinant proteins with the MBS element probe of the *FtCES18* promoter. (G) Dual‐luciferase reporter assay evaluating the activation of the *FtCES18* promoter by the cultivated and wild FtMYB32 proteins in *N. benthamiana* leaves. Data are presented as mean ± SD. Asterisks denote significant differences compared with the PK control (*P < 0.05, **P < 0.01; Student's t‐test).

It is well‐established that the N terminus of MYB transcription factors typically harbors the conserved DNA‐binding domain (DBD), whereas the C terminus dictates transactivation activity. Therefore, we hypothesized that this C‐terminal truncation specifically impairs the transcriptional efficacy of FtMYB32. Indeed, electrophoretic mobility shift assays (EMSA) conducted with identical concentrations of proteins and probes confirmed that, owing to their intact N‐terminal DBDs, both the full‐length wild‐type and the truncated cultivated FtMYB32 proteins could physically bind to the MBS element of the *FtCES18* promoter in vitro, although the wild‐type FtMYB32 exhibited a substantially stronger binding affinity (Figure 7F). Similarly, dual‐luciferase reporter assays *in planta* demonstrated that the wild‐type FtMYB32 possesses a remarkably stronger transactivation capacity toward the *FtCES18* promoter than the truncated variant (Figure 7G). Collectively, these results reveal that the missing 30 amino acids at the C terminus are indispensable for the full transcriptional activation function of FtMYB32. This domestication‐associated structural variation provides mechanistic insights into why cultivated Tartary buckwheat generally exhibits compromised basal disease resistance compared to its wild ancestors, likely reflecting a classic “growth‐defense trade‐off” driven by artificial selection for higher yields during domestication.

### A proposed working model for the *FtMYB32‐FtCES18* module in regulating Tartary buckwheat resistance against *R. Solani*

Based on the comprehensive molecular interaction and population genetics evidence, we propose a working model for the *FtMYB32‐FtCES18* regulatory module conferring *R. solani* resistance in Tartary buckwheat (Figure 8). Upon pathogen infection, the core transcription factor FtMYB32 specifically binds to the conserved MBS *cis*‐element (CAACTG) in the promoter of the downstream effector gene *FtCES18*, thereby activating its transcription. Subsequently, the highly expressed FtCES18 hydrolase efficiently catalyzes the conversion of relatively inactive MeJA into free JA, triggering substantial endogenous JA accumulation to launch downstream JA‐Ile signaling. This core hormone signaling cascade further drives the biosynthesis of downstream defensive secondary metabolites, predominantly flavonoids, ultimately establishing a high physiological frontline against *R. solani*.

**Figure 8:**
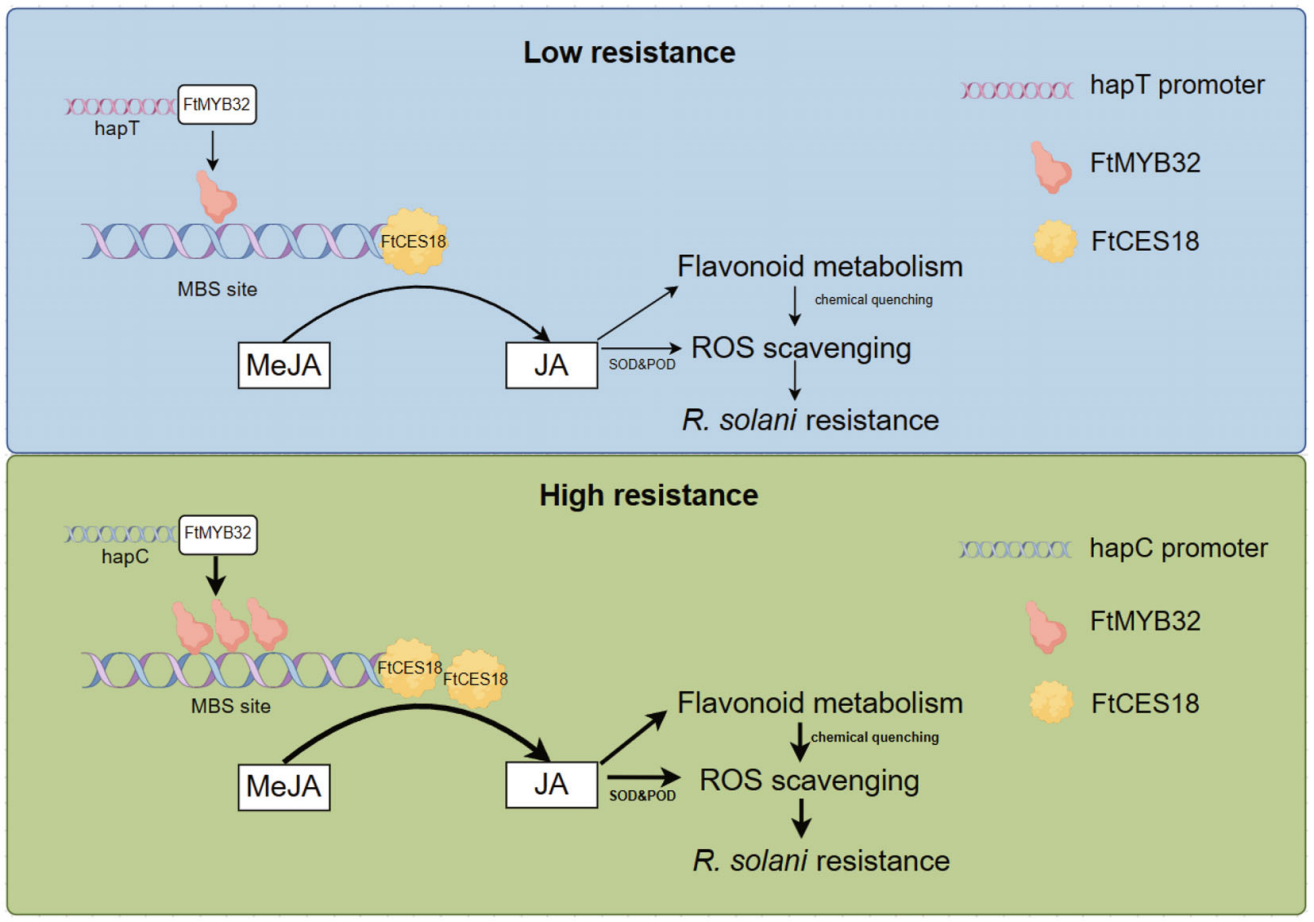
A proposed working model for the *FtMYB32*‐*FtCES18* regulatory module in mediating *R. solani* resistance in Tartary buckwheat. In low‐resistance accessions (upper panel), the hapT promoter confers weak transcriptional activity, resulting in limited FtMYB32 protein accumulation. Consequently, the weak activation of *FtCES18* leads to low hydrolase abundance, which is insufficient to trigger a robust conversion of MeJA to free JA. The resulting weak JA signaling fails to adequately activate both enzymatic antioxidant machineries (via SOD and POD) and chemical quenching pathways (via flavonoid metabolism), leading to compromised ROS scavenging and a susceptible phenotype. Conversely, in high‐resistance accessions (lower panel), the superior hapC promoter significantly elevates the basal expression of *FtMYB32*. The highly abundant FtMYB32 robustly binds to the MBS cis‐element to hyperactivate *FtCES18* transcription. The massive accumulation of *FtCES18* efficiently catalyzes MeJA hydrolysis, triggering a strong JA burst (indicated by the thick arrows). This hormone cascade profoundly and parallelly coordinates enzymatic ROS scavenging (mediated by SOD and POD) and chemical quenching (mediated by defensive flavonoid accumulation), which synergistically detoxify pathogen‐induced ROS and ultimately confers superior disease resistance.

Crucially, the natural variation (T/C transition) in the *FtMYB32* promoter among Tartary buckwheat populations directly governs the magnitude of this defense network response. Compared to the susceptible haplotype hapT, Tartary buckwheat accessions harboring the favorable haplotype hapC possess significantly stronger basal and activated transcriptional activity of *FtMYB32*. This genetic advantage enables them to more rapidly and abundantly produce FtMYB32 protein upon pathogen invasion, thereby more efficiently activating the downstream “FtCES18‐JA‐flavonoid” defense cascade. This comprehensive model fundamentally elucidates the molecular and genetic basis underlying the superior disease resistance phenotype of hapC accessions, providing profound theoretical insights and promising genetic targets for the future breeding of novel Tartary buckwheat varieties with high disease resistance.

## DISCUSSION

*R. solani* poses a major threat to Tartary buckwheat production, severely limiting its high yield and superior quality (He et al., 2023). Identifying disease‐resistance genes in Tartary buckwheat is of great significance for the development of its industry (Guan et al., 2024). Deciphering the defensive networks of crops against necrotrophic soil‐borne fungi has always been a major challenge in the fields of plant pathology and resistance breeding (Wibberg et al., 2013). The disease resistance of Tartary buckwheat is a typical quantitative trait, and GWAS provides an effective method for identifying disease resistance genes (Li et al., 2017; X. Yang, Zhang, et al., 2025). In this study, through the disease resistance evaluation of 320 Tartary buckwheat core germplasm resources combined with resequencing data, we identified a significant SNP locus and 30 candidate genes (Figure 1A, Table S1). GO classification of these genes showed that most of them are involved in cellular and metabolic processes (Figure S1a); KEGG pathway classification indicated that these genes predominantly cluster into the “plant‐pathogen interaction” and “plant hormone signal transduction” pathways (Figure S1b) (Mutuku & Nose, 2012; Rojas et al., 2014). This indicates that the disease resistance‐associated genomic region identified in this study possesses excellent reliability. Furthermore, we pinpointed *FtCES18* by combining the *R. solani* interaction transcriptome and the JA transcriptome. Elucidating the functional characteristics of *FtCES18* not only helps us understand the disease resistance mechanism of Tartary buckwheat but also contributes to the breeding of disease‐resistant varieties.

After establishing *FtCES18* as the core effector gene, we investigated its biochemical function and physiological resistance mechanisms. As a carboxylesterase, FtCES18 possesses a catalytic pocket specifically tailored for MeJA (Figure 3C). Previous studies have established that MES/CES‐mediated demethylation (hydrolysis) of MeJA acts as a critical biochemical step for activating the endogenous highly active JA signaling cascade in *Arabidopsis* and *Nicotiana attenuata*. For instance, AtMES10 in *Arabidopsis* has been confirmed to possess specific MeJA hydrolytic activity, which maintains JA homeostasis and enhances disease resistance by hydrolyzing MeJA into free JA (Wu et al., 2008; Yang et al., 2008). Furthermore, Staswick et al., through their pioneering work on the *Arabidopsis jar1* mutant, explicitly pointed out that exogenously applied MeJA must first be demethylated (i.e., hydrolyzed to JA) and subsequently conjugated to isoleucine (Ile) to become a bioactive signaling molecule (Staswick et al., 1992; Staswick & Tiryaki, 2004). Consistent with this paradigm, our *in vivo* metabolite quantification demonstrated that overexpressing *FtCES18* in *Arabidopsis* and Tartary buckwheat hairy root lines resulted in a highly significant increase in endogenous JA content (Figure 3F,H). This suggests from an *in vivo* genetic perspective that Tartary buckwheat FtCES18 equally possesses the capacity to efficiently hydrolyze MeJA. More importantly, considering that *R. solani* typically invades directly through the root system or lower leaves in contact with the soil, the specific high expression of *FtCES18* in root and leaf tissues (Figure 1F,G) provides a spatial first line of defense against pathogen invasion. In addition, necrotrophic pathogens typically hijack host machinery to intentionally trigger a destructive reactive oxygen species (ROS) burst during the early stages of infection. To counteract this lethal virulence strategy, our physiological, biochemical, and *in situ* DAB staining evidence demonstrated that *FtCES18* overexpression rapidly elevates JA levels during the infection phase, thereby enhancing the responsiveness of the host antioxidant enzyme system (POD and SOD) (Figure 2A,C–E). This pathway effectively blocks irreversible lipid peroxidation (significantly decreased MDA, Figure 2C) and pathological cell necrosis. This mechanism of rebalancing the host redox homeostasis contributes to effective defense against necrotrophic fungi.

Beyond triggering the physiological antioxidant enzyme system, the reprogramming of hormone‐driven defensive secondary metabolism constitutes another crucial pillar for plants to establish durable disease resistance (Ding et al., 2022; Guan et al., 2024). As the central hub responding to *R. solani* infection, JA signaling can significantly induce the biosynthesis of downstream defensive secondary metabolites (Ho et al., 2020; Luo et al., 2023). For instance, in *Salvia miltiorrhiza*, JA represses the expression of *SmbHLH60*, thereby inhibiting anthocyanin biosynthesis (Liu et al., 2022); in Tartary buckwheat, JA has been shown to induce *FtBMP3* expression to promote the synthesis of flavonoids such as rutin (Ding et al., 2022). Moreover, in interaction models of apple, tomato, and tobacco with pathogens, specific flavonoid compounds have been demonstrated to directly compromise fungal cell wall integrity, significantly inhibiting their mycelial development and zoospore germination (Duan et al., 2025; Treutter, 2005). Furthermore, due to their unique molecular backbones, flavonoids are considered important non‐enzymatic antioxidants *in planta*, capable of acting directly as ROS “quenchers” (L. L. Li, Li, et al., 2023). Our experimental results also support this conclusion: targeted HPLC metabolomic profiling revealed that in *FtCES18*‐overexpressing lines, the metabolism was redirected toward the downstream flavonoid biosynthesis pathway, leading to a significant accumulation of antioxidant flavonoids (e.g., quercetin and rutin) (Figure 4C). Coupled with the rapid response of antioxidant enzymes (POD and SOD) in FtCES18‐overexpressing plants during the early stages of infection (Figure 2C–E), this indicates that the host can efficiently scavenge infection‐induced excessive ROS. In other words, the JA signaling driven by *FtCES18* not only upregulates defense‐related genes at the transcriptional level (Figure 3J–M) but also promotes the synthesis of flavonoid compounds at the metabolic level (Figure 4). These flavonoids can directly inhibit pathogen growth while effectively alleviating oxidative stress, synergistically establishing a defense network in Tartary buckwheat against *R. solani*.

Previous studies have demonstrated that the metabolism of JA and flavonoids is subject to direct or indirect regulation by transcription factors. For example, *PdMYB118* in *Populus deltoides* stimulates JA homeostasis and activates the expression of anthocyanin biosynthetic genes, such as *AGBs* (Wang et al., 2020). In Tartary buckwheat, *FtDREB2* enhances stress tolerance by promoting delphinidin synthesis (Wang et al., 2025). Consistent with these findings, our results reveal that FtMYB32 directly binds to the MBS *cis*‐elements in the *FtCES18* promoter to activate its expression (Figure 5D–E). Subsequently, FtCES18 effectively catalyzes the demethylation (hydrolysis) of relatively inactive MeJA into free JA, which is subsequently converted into active JA‐Ile to confer disease resistance. As the crucial rate‐limiting enzyme for this conversion, *FtCES18* acts as a key node connecting the hormone signaling and flavonoid regulatory networks in Tartary buckwheat. Intriguingly, population genetics analysis revealed that *FtCES18* has undergone strict purifying selection during evolution, remaining highly conserved with no significant phenotype‐associated variations in its CDS or promoter regions. However, we precisely mapped a T/C single nucleotide polymorphism (SNP) in the *FtMYB32* promoter (Figure 6A), which is significantly associated with the disease resistance phenotype (Figure 6B). Moreover, this natural variation directly stratifies the resistance hierarchy of the Tartary buckwheat population. Compared with the susceptible haplotype (hapT), accessions harboring the favorable haplotype hapC do not exhibit altered protein function; rather, they substantially elevate the basal and activated transcriptional capacity of FtMYB32 (Figure 6C–E). This “dosage fine‐tuning” at the promoter level cascades to the downstream expression of *FtCES18*, ultimately endowing hapC accessions with a stronger JA burst and stronger flavonoid biosynthesis (Figure 6F,G). Strikingly, our re‐analysis of metabolic GWAS data identified a prominent association signal for quercetin content directly adjacent to the *FtMYB32* genomic locus (Figure S13). This population‐level genetic evidence supports the conclusion that *FtMYB32* acts as a key transcriptional regulator orchestrating the downstream accumulation of defensive flavonoids. Interestingly, comparative analysis between wild and cultivated Tartary buckwheat revealed a distinct variation within the *FtMYB32* coding region. Wild Tartary buckwheat retains the full‐length, highly active FtMYB32 protein, which is capable of driving a robust *FtCES18*‐JA defense cascade. However, hyperactive defense responses can incur substantial metabolic costs. In cultivated accessions, the naturally occurring C‐terminal truncation of FtMYB32 significantly attenuates its transactivation activity. This variation likely represents an adaptive selection during domestication, allowing cultivated Tartary buckwheat to dampen the excessive metabolic penalty of severe JA bursts while maintaining a baseline immune response.

In conclusion, our findings delineate a hierarchical regulatory module that links transcriptional control, hormonal activation, and metabolic output to confer *R. solani* resistance in Tartary buckwheat (Figure 8). The *FtMYB32*‐*FtCES18* module effectively bridges the pathogen‐induced JA burst with the accumulation of downstream defensive flavonoids. Importantly, this study highlights how cis‐regulatory polymorphisms and domestication‐associated structural variations synergistically drive phenotypic diversification in plant immunity. Collectively, elucidating this multi‐dimensional defense network provides elite genetic targets and a solid theoretical foundation for breeding novel Tartary buckwheat cultivars enriched in bioactive compounds and possessing disease resistance.

## MATERIALS AND METHODS

### Plant materials

The *F. tataricum* accessions employed in this study were kindly provided by the Buckwheat Germplasm Resources Group, Institute of Crop Sciences, Chinese Academy of Agricultural Sciences (CAAS). Plant cultivation and subsequent management were conducted in accordance with the protocols established by Zhang (Zhang et al., 2021). Furthermore, *Arabidopsis thaliana* and *N. benthamiana* were grown in a climate‐controlled greenhouse with a relative humidity range of 65–70%. The environmental parameters, including a 16‐h light/8‐h dark photoperiod, were strictly maintained as previously described by Zhao (H. Zhao, He, et al., 2023). The incubator used was Model TCC800 (Fujian Jiupo Biotechnology Co., Ltd., Fuzhou, China).

### GWAS and identification of candidate genes

Resequencing data for the *F. tataricum* accessions were derived from previously published research (Zhang et al., 2021). By integrating the disease resistance indices of 320 accessions, a GWAS was conducted according to the methodology described by He et al. (2023). Manhattan plots were subsequently generated using the R software. Genes located within a 100‐kb window upstream and downstream of the significant loci were designated as potential candidates associated with disease resistance. Furthermore, the expression profiles of these candidates were analyzed using transcriptome data from Tartary buckwheat challenged with *R. solani*. Ultimately, specific candidate genes were identified and selected for subsequent functional characterization.

### Linkage disequilibrium (LD) block

Based on the lead SNPs identified via GWAS, SNP genotype data within a 100‐kb window upstream and downstream of the target loci were extracted. Linkage disequilibrium (LD) parameters (r^2^) between pairs of SNPs were calculated using PLINK software (Purcell et al., 2007). LD block heatmaps and haplotype structures were generated and visualized using LDBlockShow or Haploview. Haplotype blocks within the candidate regions were defined based on the magnitude of LD decay. Subsequently, potential candidate genes located within these high‐LD blocks were prioritized for further functional validation based on genomic annotations.

### Haplotype analysis

Haplotype analysis of candidate genes was performed using CandiHap software by integrating the genomic resequencing data and disease resistance indices of 320 Tartary buckwheat accessions (X. Li, Shi, et al., 2023). The analytical region was defined as the 2000‐bp promoter sequence upstream of the start codon (ATG) and the entire coding DNA sequence (CDS). Significant differences in disease resistance among the identified haplotypes were evaluated using appropriate statistical tests to determine the phenotypic effects of different allelic variations.

### RNA extraction and RT‐qPCR

For gene expression analysis, leaves were harvested from two‐week‐old wild‐type (WT) and overexpression (OE) *Arabidopsis* plants grown under a 22–25°C temperature regime and a 16‐h light/8‐h dark photoperiod. The collected tissues were immediately frozen in liquid nitrogen and stored at −80°C until further use. Total RNA was extracted using the RNA Easy Fast Plant Tissue RNA Extraction Kit (DP452, Tiangen), and first‐strand cDNA was synthesized using the HiScript III First Strand cDNA Synthesis Kit with gDNA wiper (R323‐01, Vazyme). The relative expression levels of target genes were quantified via quantitative real‐time PCR (RT‐qPCR) and calculated using the 2^−ΔΔCT^ method. For Tartary buckwheat, samples were collected at 0, 3, 6, and 24 h post‐inoculation with *R. solani*, following the same RT‐qPCR procedures as described above. All primers utilized in this study are listed in Table S13.

### Transcriptome analysis

The time‐course *R. solani* interaction transcriptome and the methyl jasmonate (MeJA) treatment transcriptome datasets analyzed in this study were obtained from our previous study (He et al., 2023) and are publicly available under BioProject accession number PRJNA917064. Briefly, for the *R. solani* infection assay, surface‐sterilized Tartary buckwheat seeds were cultivated at 22°C for 21 days. Seedling leaves were subsequently inoculated with *R. solani* mycelia subcultured on Potato Dextrose Agar (PDA) medium. Inoculated leaf samples were harvested at 0, 6, 14, and 22 h post‐inoculation (hpi). For the MeJA treatment, leaves of 21‐day‐old Tartary buckwheat seedlings were treated with 50 μM MeJA (W341002, Sigma‐Aldrich, Taufkirchen, Germany) and harvested at 1, 4, or 12 h post‐treatment.

For both datasets, three independent biological replicates were performed, with each replicate obtained by pooling leaf tissues from 10 individual seedlings. Total RNA was extracted using TRIzol reagent (15 596 026, Invitrogen, Carlsbad, USA), and residual genomic DNA was eliminated using DNase I (2270A, TaKaRa, Kusatsu, Japan). RNA‐seq libraries were constructed using the TruSeq RNA Sample Prep Kit (RS‐122‐2001, Illumina, San Diego, USA) and sequenced on the Illumina HiSeq X Ten/NovaSeq 6000 platform. Clean reads were aligned to the Tartary buckwheat HERA reference genome using HISAT2 (Kim et al., 2015), and differential expression analysis was performed using the DESeq2 and DEGseq (Love et al., 2014; Wang et al., 2010).

### Cloning and vector construction of the *FtCES18* gene

The full‐length coding sequence (CDS) of *FtCES18* was cloned into the overexpression vector using the homologous recombination method. Detailed information regarding the specific primers and restriction enzyme sites is provided in Table S13. The procedures for vector construction and subsequent genetic transformation were performed as previously described by our laboratory (He et al., 2024). The overexpression construct for *FtMYB32* was generated following the same methodology as described above.

### Generation of transgenic lines

*Arabidopsis* transformation was performed via the floral dip method using *Agrobacterium tumefaciens* strain GV3101. Transgenic lines were screened on Murashige and Skoog (MS) medium supplemented with 60 μg/mL hygromycin. Three independent, stable, and high‐expressing lines from the T3 generation were selected for further analysis. Tartary buckwheat hairy roots transformation was carried out using a previously established method (He et al., 2024). Similarly, three representative high‐expressing lines were identified and used in subsequent experiments.

### GUS histochemical staining

Transgenic *Arabidopsis* tissues were submerged in GUS histochemical staining buffer (containing 1 mg/mL X‐Gluc). After vacuum infiltration, the samples were incubated at 37°C for 2 h. The stained tissues were subsequently decolorized in 75% (v/v) ethanol until the chlorophyll was thoroughly removed, then visualized under a stereomicroscope.

### Disease resistance assay of transgenic plants

The disease resistance assay in *Arabidopsis* was performed using young, healthy, and undamaged rosette leaves collected from two‐week‐old WT and *FtCES18*‐overexpressing (OE) plants. Detached leaves were placed on moist filter paper in Petri dishes, and uniformly sized agar plugs of *Rhizoctonia solani* were gently placed at the center of each leaf. The dishes were sealed and maintained at 28°C. Disease symptoms were recorded and photographed at 2 days post‐inoculation. Each genotype was evaluated using three independent biological replicates.

For histochemical detection of hydrogen peroxide (H_2_O_2_) accumulation following *R. solani* infection, young and intact rosette leaves were carefully collected to minimize mechanical damage and subjected to 3,3′‐diaminobenzidine (DAB) staining. The leaves were completely immersed in commercial DAB staining solution (Coolaber, Beijing, China) and vacuum‐infiltrated for 30 min to facilitate penetration of the staining solution into the leaf tissues. The samples were subsequently incubated in the DAB staining solution for 8 h at room temperature in the dark. After staining, the leaves were transferred to commercial decolorization solution (Coolaber, Beijing, China) and incubated at 80°C until chlorophyll was sufficiently removed. The decolorized leaves were then photographed to visualize the distribution of reddish‐brown DAB precipitates indicative of H_2_O_2_ accumulation.

### Measurement of antioxidant enzyme activities

The antioxidant enzyme activities in transgenic *Arabidopsis* plants were determined using Superoxide Dismutase (SOD) (BC0170, Solarbio, Beijing, China) and Peroxidase (POD) (BC0090, Solarbio) activity assay kits. Additionally, the malondialdehyde (MDA) content was measured to evaluate the level of lipid peroxidation using an MDA assay kit (BC0025, Solarbio). All assays were conducted following the manufacturer's instructions, with each sample analyzed in triplicate to ensure biological consistency.

### Quantification of endogenous H_2_O_2_ and O_2_ •^−^ contents

The endogenous hydrogen peroxide (H_2_O_2_) and superoxide anion (O_2_•^−^) contents were quantified using commercial assay kits (Cat# BC3595 and Cat# BC1295, respectively; Solarbio, Beijing, China). The experimental procedures were strictly performed in accordance with the manufacturer's protocols. The optical density (OD) values were measured at wavelengths of 415 nm for H_2_O_2_ and 530 nm for O_2_•^−^ using a microplate reader. Subsequently, the concentrations of H_2_O_2_ and O_2_•^−^ in the samples were calculated based on standard curves generated with known concentrations of their respective standards and normalized to tissue fresh weight.

### Quantification of JA, MeJA, and JA‐Ile content

The endogenous JA content was quantified using a plant JA enzyme‐linked immunosorbent assay (ELISA) kit (MSKBIO, Wuhan, China). The experimental procedures were strictly performed in accordance with the manufacturer's protocols. The optical density (OD) values were measured at a wavelength of 450 nm using a microplate reader. Subsequently, the JA concentrations in the samples were calculated based on a standard curve generated with known concentrations of JA.

The levels of methyl jasmonate (MeJA) and jasmonic acid‐isoleucine (JA‐Ile) were quantified using ELISA kits (YuanJu Bio, Shanghai, China). The assay was performed strictly according to the manufacturer's instructions. Optical density (OD) was measured at 450 nm using a microplate reader. Sample concentrations were calculated based on standard curves generated from known concentrations of the respective standards and normalized to tissue fresh weight.

### Virus‐induced gene silencing (VIGS) assay

The pTRV1 and pTRV2‐*FtCES18* constructs were individually transformed into *Agrobacterium tumefaciens* strain GV3101 competent cells. The transformed cultures were then inoculated at a 1:100 (v/v) ratio into 50 mL of YEB liquid medium supplemented with 100 mg/L kanamycin and 50 mg/L rifampicin, and grown at 28°C with shaking until an OD_600_ of approximately 0.8 was reached. After normalizing the optical density, *Agrobacterium* cultures containing pTRV1 and pTRV2‐*FtCES18* were mixed at a 1:1 (v/v) ratio. Acetosyringone (AS, 100 μM) was added to the mixture, which was then incubated to prepare the VIGS infiltration solution. The resulting solution was infiltrated into the leaves of Tartary buckwheat ‘ZK3’, using *Agrobacterium* harboring pTRV1 and the empty pTRV2 vector as the control. Fifteen days post‐infiltration, leaves were collected for RNA extraction to verify silencing efficiency via RT‐qPCR and subjected to disease resistance assays.

### Subcellular localizations of FtCES18

*Agrobacterium tumefaciens* strain GV3101 harboring the pCAMBIA2300‐*FtCES18* construct was inoculated into 5 mL of YEB medium supplemented with 100 μg/mL kanamycin and 50 μg/mL rifampicin. The culture was incubated at 22°C with shaking at 220 rpm until an OD_600_ of 0.8–1.2 was achieved. Cells were harvested by centrifugation for 15 min and resuspended in infiltration buffer (10 mmol/L MgCl_2_, 10 mmol/L MES, and 200 μmol/L acetosyringone) to a final OD_600_ of 0.8. The suspension was then infiltrated into the abaxial surface of tobacco (*N. benthamiana*) leaves using a needleless syringe. Following 72 h of incubation in the dark, green fluorescent protein (GFP) signals were visualized using a laser scanning confocal microscope (ZEISS LSM 900). The excitation and emission wavelengths were set at 488 nm and 561 nm, respectively.

### Phylogenetic tree construction of FtCES18

To investigate the evolutionary relationship of CES18, homologous protein sequences were retrieved from the NCBI database using the BLASTP program with default parameters. Multiple sequence alignment was performed using the ClustalW algorithm implemented in MEGA software. A phylogenetic tree was constructed using the Neighbor‐Joining (NJ) method with 1000 bootstrap replicates to evaluate branch support. All other parameters were set to default values.

### Molecular docking analysis

The three‐dimensional protein model was first predicted using AlphaFold3, and the corresponding ligand structures in SDF format were retrieved from the PubChem database. Preprocessing of both the protein and ligands was conducted using the Molecular Operating Environment (MOE) software. Specifically, the QuickPrep module was employed for structural correction, protonation, removal of unbound water molecules, and energy minimization. Following protein preparation, potential active sites were identified to determine the approximate binding pocket. A docking box was then defined, centered at the predicted pocket coordinates, with its dimensions and parameters optimized based on the ligand properties. Finally, the docking precision and parameters were configured, and the molecular docking simulations were initiated.

### Determination of flavonoid profiles via HPLC‐QQQ

Sample preparation: Lyophilized tissues were homogenized into a fine powder, and 0.1 g aliquots was subjected to ultrasound‐assisted extraction using 10 mL of 80% (v/v) aqueous methanol. The extraction was maintained at 50°C for 40 min. Following centrifugation, the resulting supernatants were purified using 0.22 μm pore‐size membranes and transferred into autosampler vials for downstream quantification. Chromatographic Separation: Flavonoid separation was achieved on an Agilent SB‐C18 column (2.1 mm × 75 mm, 2.7 μm) maintained at 40°C. The mobile phase comprised 0.1% formic acid in water (Solvent A) and 0.1% formic acid in acetonitrile (Solvent B), delivered at a constant flow rate of 0.35 mL/min. The optimized gradient program was executed as follows: 0 min, 5% B; 0–9 min, a linear ramp to 95% B; 9–10 min, isocratic 95% B; 10–11 min, rapid return to 5% B; and 11–14 min, column re‐equilibration. The sample injection volume was 2 μL. Mass Spectrometry Parameters: Detection was performed using a triple quadrupole (QQQ) mass spectrometer in electrospray ionization (ESI) mode. Nitrogen was utilized as both the drying and nebulizing gas, with the temperature and flow rate fixed at 300°C and 8.0 L/min, respectively. The ionization parameters were calibrated as follows: nebulizer pressure, 35 psi; capillary voltage, 3.5 kV; fragmentor voltage, 175 V; and skimmer voltage, 65 V. Collision energies (CE) ranging from 10 to 50 V were applied for fragmentation, with data acquisition spanning a mass‐to‐charge (*m/z*) range of 50–1000.

### Transcriptomic clustering analysis

The time‐course transcriptomic datasets derived from the *F. tataricum*–*R. solani* interaction were formatted and analyzed using the TCseq package in R. After data normalization and quality filtering, all identified differentially expressed genes (DEGs) were partitioned into 15 distinct clusters based on their temporal expression profiles. This clustering was performed to categorize genes sharing similar kinetic patterns, facilitating the identification of key regulatory modules involved in the plant's response to fungal infection.

### *In vitro* enzyme activity assay of FtCES18

To determine the enzymatic activity of FtCES18 toward methyl jasmonate (MeJA), recombinant GST‐FtCES18 fusion protein was heterologously expressed in *Escherichia coli* BL21 (DE3) and purified by affinity chromatography. The enzymatic reactions were performed in a total volume of 100 μL containing 50 mM Tris–HCl (pH 7.5), 2 mM DTT, 1 mM MeJA (Sigma‐Aldrich, USA, St. Louis, MO, USA), and either 2 or 5 μg of purified GST‐FtCES18 protein. Reactions containing purified GST protein and MeJA under identical conditions were used as negative controls. The reactions were initiated by the addition of MeJA and incubated at 30°C for 30 min. Enzymatic reactions were terminated by adding 50 μL of ice‐cold LC–MS‐grade acetonitrile. After vortex mixing, the samples were centrifuged at 14000 × ***g*** for 15 min at 4°C to remove precipitated proteins. The resulting supernatants were passed through a 0.22‐μm organic membrane filter and subjected to LC–MS/MS analysis.

Chromatographic separation was performed on a C8 reversed‐phase column using an optimized LC method. Jasmonic acid (JA) was analyzed using a triple‐quadrupole LC–MS/MS system (Shimadzu, Kyoto, Japan) operated in negative electrospray ionization (ESI−) mode. For multiple reaction monitoring (MRM), the transition of JA was monitored at *m/z* 209.20 → 109.00 with a collision energy of 19.0 eV. An authentic JA standard (Sigma‐Aldrich, USA) was analyzed under identical chromatographic and mass‐spectrometric conditions to determine the retention time and confirm the chromatographic identity of the enzymatic product. For further structural confirmation, MS/MS product‐ion scanning was performed using the deprotonated JA precursor ion at *m/z* 209.20 ([M − H]^−^) with a collision energy of 25.0 eV. The fragmentation pattern of the enzymatic product was compared with that of the authentic JA standard obtained under identical conditions. Three independent enzymatic reactions were performed for each treatment.

### *Cis*‐acting regulatory element prediction

The 2000‐bp genomic sequence upstream of the start codon (ATG) for the target gene was retrieved from the Tartary Buckwheat Genome Database to serve as the predicted promoter region. This sequence was subsequently submitted to the PlantCARE online database for the identification of *cis*‐acting regulatory elements. Based on the functional annotation, motifs associated with biotic and abiotic stress responses, as well as phytohormone signaling—such as ABRE (abscisic acid responsiveness), GARE (gibberellin‐responsiveness), TCA‐element (salicylic acid responsiveness), and CGTCA‐motif (MeJA‐responsiveness)—were prioritized for analysis. Finally, the spatial distribution of these key elements within the promoter region was visualized using TBtools software (Chen et al., 2023).

### Yeast one‐hybrid (Y1H) assay

To verify the physical interaction between the FtMYB32 protein and the *FtCES18* promoter, a yeast one‐hybrid (Y1H) assay was performed. The full‐length coding sequence (CDS) of *FtMYB32* was amplified and ligated into the pGADT7 vector to generate the prey construct (AD‐FtMYB32). Concurrently, the promoter fragment of *FtCES18* was cloned into the pHIS2 reporter vector. Both recombinant plasmids were co‐transformed into the yeast strain Y187 using the lithium acetate method. Transformants were initially screened on double‐dropout medium (SD/−Trp/−Leu). To assess the protein–DNA interaction, positive single colonies were selected and streaked onto triple‐dropout medium (SD/−Trp/−Leu/−His) supplemented with an optimal concentration of 3‐amino‐1,2,4‐triazole (3‐AT) to suppress auto‐activation. Detailed procedures were conducted as previously described in our laboratory's research (He et al., 2024).

### Electrophoretic mobility shift assay

The CDS of *FtMYB32* was cloned into a GST‐fusion vector and transformed into *Escherichia coli* strain BL21 (DE3) for prokaryotic protein expression and purification. Biotin‐labeled DNA probes containing the MBS *cis*‐acting element from the *FtCES18* promoter were synthesized (Sangon Biotech; detailed probe information is provided in Table S13). The purified GST‐FtMYB32 fusion protein (with GST protein used as a negative control) was incubated with the labeled probes. The EMSA was conducted using the LightShift EMSA Optimization & Control Kit (Thermo Fisher Scientific, Waltham, MA, USA) according to the manufacturer's instructions.

### Dual‐luciferase reporter assay

To evaluate the transcriptional regulation of *FtCES18* by FtMYB32, the *FtCES18* promoter fragment was cloned into the pGreen II 0800‐LUC reporter vector, while the full‐length CDS of *FtMYB32* was inserted into the pGreen II 62‐SK effector vector. Primers used for construct generation are provided in Table S13. Both recombinant plasmids were transformed into *Agrobacterium tumefaciens* strain GV3101. For transient expression assays, the *Agrobacterium* cultures harboring the effector and reporter constructs were mixed and co‐infiltrated into the leaves of four‐week‐old *N. benthamiana* plants. After 48 h of incubation in the dark, the leaves were treated with potassium luciferin. Following 5 min of dark adaptation, bioluminescence signals were captured using an *in vivo* plant imaging system. Finally, leaf discs were collected to quantify firefly luciferase (LUC) and Renilla luciferase (REN) activities using a dual‐luciferase assay kit (DL101, Vazyme), with the LUC/REN ratio used to determine transcriptional activation.

### Chromatin immunoprecipitation (ChIP)‐qPCR assay

ChIP was performed using freshly cultured hairy root tissues from the control line (A4) and the FtMYB32‐overexpressing line (1307‐FtMYB32). Tissues were cross‐linked in fix buffer containing 1% (v/v) formaldehyde under vacuum for 10 min, followed by the addition of 2.5 M glycine (0.125 M final concentration) for 5 min to terminate the cross‐linking reaction. Fixed tissues were thoroughly washed with deionized water and ground into a fine powder in liquid nitrogen. The ground powder was lysed with 300 μL Buffer S (50 mM HEPES‐KOH pH 7.5, 150 mM NaCl, 1 mM EDTA, 1% Triton X‐100, 0.1% sodium deoxycholate, 1% SDS, and 1 mM PMSF) at 4°C for 10 min. The homogenate was mixed with 1.2 mL Buffer F (50 mM HEPES‐KOH pH 7.5, 150 mM NaCl, 1 mM EDTA, 1% Triton X‐100, 0.1% sodium deoxycholate, and 1 mM PMSF), and chromatin was sheared into 250–600 bp DNA fragments using a Bioruptor Plus sonication system (Diagenode, Liège, Belgium). The lysate was centrifuged at 20 000 ***g*** for 10 min at 4°C, and an aliquot of the supernatant was collected as the Input control. Subsequently, the remaining supernatant was incubated with anti‐MYC magnetic beads at 4°C overnight.

The immunoprecipitated chromatin was washed sequentially with low‐salt ChIP buffer (50 mM HEPES‐KOH, 150 mM NaCl, 1 mM EDTA, 1% Triton X‐100, 0.1% sodium deoxycholate, and 0.1% SDS), high‐salt ChIP buffer (low‐salt ChIP buffer with 150 mM NaCl replaced by 350 mM NaCl), ChIP wash buffer (10 mM Tris–HCl pH 8.0, 250 mM LiCl, 0.5% NP‐40, 1 mM EDTA, and 0.1% sodium deoxycholate), and TE buffer. Protein–DNA complexes were eluted from the beads using freshly prepared ChIP elution buffer (1% SDS, 0.1 M NaHCO3) at 65°C for 15 min. The eluate was mixed with 0.2 M NaCl and incubated at 65°C for 10 h for reverse cross‐linking, followed by proteinase K digestion at 45°C for 2 h. DNA was purified using a commercial kit (NEB, Cat. No. T1130S). The purified DNA (ChIP and Input samples from both A4 control and 1307‐FtMYB32 lines) was subjected to qPCR analysis. To evaluate binding specificity, two sets of primers were used: one targeted the MBS element‐containing region on the *FtCES18* promoter, and the other targeted an adjacent non‐binding negative control region. Relative enrichment was quantified using the % Input method.

### Statistical analysis

All experiments in this study were independently conducted with at least three biological replicates, and quantitative data are presented as the mean ± standard deviation (SD). Statistical analyses and figure plotting were performed using the GraphPad Prism 8.0.2 software (GraphPad Software, San Diego, CA, USA).

For comparisons between two experimental groups (e.g., ChIP‐qPCR enrichment assays, dual‐luciferase reporter assays, and pairwise gene expression comparisons between transgenic lines and controls), statistical significance was determined using two‐tailed unpaired Student's t‐test. For comparisons among multiple groups (e.g., lesion areas, time‐course expression profiles, physiological enzyme activities, ROS/MDA contents, and metabolite abundances across different lines), one‐way analysis of variance (ANOVA) followed by Tukey's post hoc test or Duncan's multiple range test was applied. Differences were considered statistically significant at *P* < 0.05 (* or distinct lowercase letters) and highly significant at *P* < 0.01 (**).

## AUTHOR CONTRIBUTIONS

MZ, CG, and ZF conceived and designed the work. YC and YL performed the experiments, and YC, YL, NC, and CG analyzed the data. YC and YL wrote the manuscript. MZ, CG, HW, JCR, MDL, and XP revised the manuscript. All authors have read and agreed to the published version of the manuscript.

## CONFLICT OF INTEREST

The authors declare no conflicts of interest.

## Supporting information


**Figure S1.** Functional classification of the candidate genes within the GWAS significant locus. (a) Gene Ontology (GO) classification analysis of the 30 annotated candidate genes located within the linkage disequilibrium (LD) block on Ft8. The results indicate that the majority of these genes are concentrated in cellular and metabolic processes. (b) KEGG pathway classification of candidate genes. The genes are primarily include in immune‐related pathways, specifically “plant‐pathogen interaction” and “plant hormone signal transduction.”
**Figure S2.** Expression profile of FtCES18 in response to *R. solani* infection. The temporal expression levels of FtCES18 in Tartary buckwheat at different time points post‐inoculation with *R. solani*, as derived from the interaction transcriptome data (*P < 0.05, **P < 0.01; Student's t‐test).
**Figure S3.** Time‐course expression profiles of four candidate genes under *R. solani* challenge and mock conditions. (a–d) Relative transcript abundance of four candidate genes—(a) *FtPinG0809058500.01*, (b) *FtPinG0809059200.01*, (c) *FtPinG0809059600.01*, and (d) *FtPinG0809060200.01*—in Tartary buckwheat leaves inoculated with *R. solani* (RS treated) or blank agar plugs (Mock) across 0, 6, 14, and 22 h post‐inoculation (hpi), as quantified by RT‐qPCR. Expression levels of each gene at 0 h were set to 1.0 for normalization. Data are presented as mean ± SD of three independent biological replicates. Asterisks denote statistically significant differences compared with the corresponding 0 h control (*P < 0.05, **P < 0.01; ns, not significant; Student's t test).
**Figure S4.** Relative expression levels of FtCES18 in different transgenic lines. (a) Relative expression levels of FtCES18 in wild‐type (WT) and three independent FtCES18‐overexpressing (OE1, OE2, OE3) Arabidopsis lines. (b) Relative expression levels of FtCES18 in the Mock control and FtCES18‐silenced (VIGS#1, VIGS#2, VIGS#3) Tartary buckwheat leaves. Data are presented as mean ± SD from three independent biological replicates (*P < 0.05, **P < 0.01; Student's t‐test).
**Figure S5.** Western blot analysis of purified GST‐FtCES18 protein. M: M5 Prestained Protein Ladder (10–180 kDa).
**Figure S6.** LC–MS/MS product ion spectrum of the enzymatic reaction product generated by recombinant FtCES18. Representative MS/MS spectrum (product ion scan) of the precursor ion at m/z 209.15 ([M‐H]‐ of JA) acquired under negative electrospray ionization (ESI‐) mode with a collision energy of 25.0 eV. The characteristic product ions at m/z 59.05 (base peak) and m/z 109.00 confirm the structural identity of the reaction product as free JA.
**Figure S7.** Relative expression levels of FtCES18 in transgenic hairy roots. Relative expression levels of FtCES18 in wild‐type and three independent FtCES18‐overexpressing (OE1, OE2, OE3) Tartary buckwheat hairy root lines. Data are presented as mean ± SD from three independent biological replicates (*P < 0.05, **P < 0.01; Student's t‐test).
**Figure S8.** Quantitative standard curves for MeJA (a), JA (b), and JA‐Ile (c). These standard curves were generated using standard samples from an enzyme‐linked immunosorbent assay (ELISA) kit.
**Figure S9.** Endogenous MeJA and JA‐Ile contents in FtCES18 overexpressing and FtCES18‐silenced Tartary buckwheat lines. (a) Endogenous MeJA content and (b) JA‐Ile content in wild‐type control (A4) and three independent FtCES18 overexpressing Tartary buckwheat hairy root lines. (c) Endogenous MeJA content and (d) JA‐Ile content in control (Mock) and three independent FtCES18‐silenced Tartary buckwheat leaves. Data in bar graphs are presented as mean ± SD of three independent biological replicates. Asterisks denote statistically significant differences compared with the respective controls (A4 for hairy roots, Mock for VIGS leaves) (*P < 0.05, **P < 0.01; Student's t‐test).
**Figure S10.** A simplified schematic of the JA biosynthesis and signaling pathway. The diagram illustrates the conversion of key metabolites and the specific steps catalyzed by the genes evaluated in this study, including AtLOX2, FtCES18, AtJAR1, and the downstream responsive genes AtJAZ1 and AtPDF1.2. 12 OPDA, 12‐oxophytodienoic acid; MeJA. Methyl jasmonate; JA‐Ile, Jasmonoyl isoleucine; LOX2, Lipoxygenase; JAR1, Jasmonate Resistant 1; JAZ1, Jasmonate ZIM‐domain protein; PDF1.2, Plant Defensin 1.2.
**Figure S11.** Standard curve for total flavonoid quantification. The standard curve was generated to determine the total flavonoid concentrations in the samples. The linear regression equation of the standard curve is y = 2.5839x – 0.1388 (R2 = 0.9991).
**Figure S12.** Gene Ontology (GO) classification of co expressed genes in Cluster 8. The analysis reveals that the genes within the *R. solani*‐induced Cluster 8 module are broadly involved in core biological processes, including “metabolic process” and “response to stimulus”.
**Figure S13.** GWAS signals for quercetin content trait. (a) Manhattan plot displaying the significant SNP signals associated with quercetin content. The red arrow indicates the genomic locus containing FtMYB32. The red dashed line represents the genome‐wide significance threshold. (b) Quantile quantile (Q‐Q) plot for the GWAS of quercetin content.
**Figure S14.** Relative transcript abundance of FtMYB32 and downstream FtCES18 in control (TRV) and three independent FtMYB32‐silenced (FtMYB32‐VIGS) Tartary buckwheat leaves. Asterisks denote statistically significant differences compared with the TRV control (Student's t‐test; **P < 0.01).
**Figure S15.** Relative transcript abundance of key flavonoid biosynthetic structural genes, including flavanone 3‐hydroxylase (FtF3H) (a), flavonoid 3′‐hydroxylase (FtF3′H) (b), flavonol synthase (FtFLS) (c), and rutin rhamnosyltransferase 1 (FtRT1) (d), in control (A4) and three independent FtMYB32‐overexpressing (FtMYB32‐OE) Tartary buckwheat hairy root lines. Data are presented as mean ± SD of three independent biological replicates. Asterisks denote statistically significant differences compared with the A4 control (*P < 0.05, **P < 0.01; Student's‐test).
**Figure S16.** Transient expression of FtMYB32 enhances resistance to *R. solani* in *N. benthamiana* leaves. The left half of each leaf was agroinfiltrated with an empty vector (EV) control, while the right half was infiltrated with the 1307 FtMYB32 overexpression construct.
**Figure S17.** Relative expression levels of FtMYB32 and FtCES18 in Tartary buckwheat accessions with different haplotypes. (a) Relative expression levels of FtMYB32 in representative accessions carrying the hapT or hapC haplotype. (b) Relative expression levels of FtCES18 in the corresponding accessions. Data are presented as mean ± SD from three independent biological replicates.

**Table S1.** List of candidate genes associated with *R. solani* resistance identified by GWAS.
**Table S2.** Expression profiles of GWAS‐identified candidate genes in the *R. solani* interaction transcriptome.
**Table S3.** Expression profiles of GWAS‐identified candidate genes in the jasmonic acid (JA)‐treated transcriptome.
**Table S4.** Physiological and biochemical parameters in wild‐type and FtCES18‐overexpressing Arabidopsis before and after *R. solani* infection.
**Table S5.** Quantification of MeJA, JA and JA‐Ile content in wild‐type and FtCES18‐overexpressing Arabidopsis at different time points post‐inoculation with *R. solani*.
**Table S6.** Quantification of MeJA, JA and JA‐Ile content in wild‐type and FtCES18‐overexpressing Tartary buckwheat hairy roots.
**Table S7.** Quantification of MeJA, JA and JA‐Ile content in control (Mock) and FtCES18‐silenced (VIGS) Tartary buckwheat leaves.
**Table S8.** Expression profiles of flavonoid biosynthesis pathway genes in the jasmonic acid (JA)‐treated transcriptome.
**Table S9.** Quantification of flavonoid contents (μg/g) in wild‐type and FtCES18‐overexpressing Tartary buckwheat hairy roots via HPLC.
**Table S10.** List of genes in Cluster 8 from the *R. solani* interaction transcriptome.
**Table S11.** Analysis of cis‐acting regulatory elements in the FtCES18 promoter.
**Table S12.** Quantification of jasmonic acid (JA) content (pmol/L) in Tartary buckwheat accessions with different FtMYB32 haplotypes.
**Table S13.** Sequences of primers used in this study.

## Data Availability

The data that support the findings of this study are available on request from the corresponding author. The data are not publicly available due to privacy or ethical restrictions.
