# Supplementary material for: Natural variation in a FtMYB32 ‐ FtCES18 module drives Rhizoctonia solani resistance by reprogramming jasmonate and flavonoid metabolism in Tartary buckwheat
Source: Plant J. 2026 Oct 26;128(2):e71176. doi: 10.1111/tpj.71176 (PMC13698632; doi:10.1111/tpj.71176)
Supplement: Supplementary file 1 [file TPJ-128-0-s002.pdf]

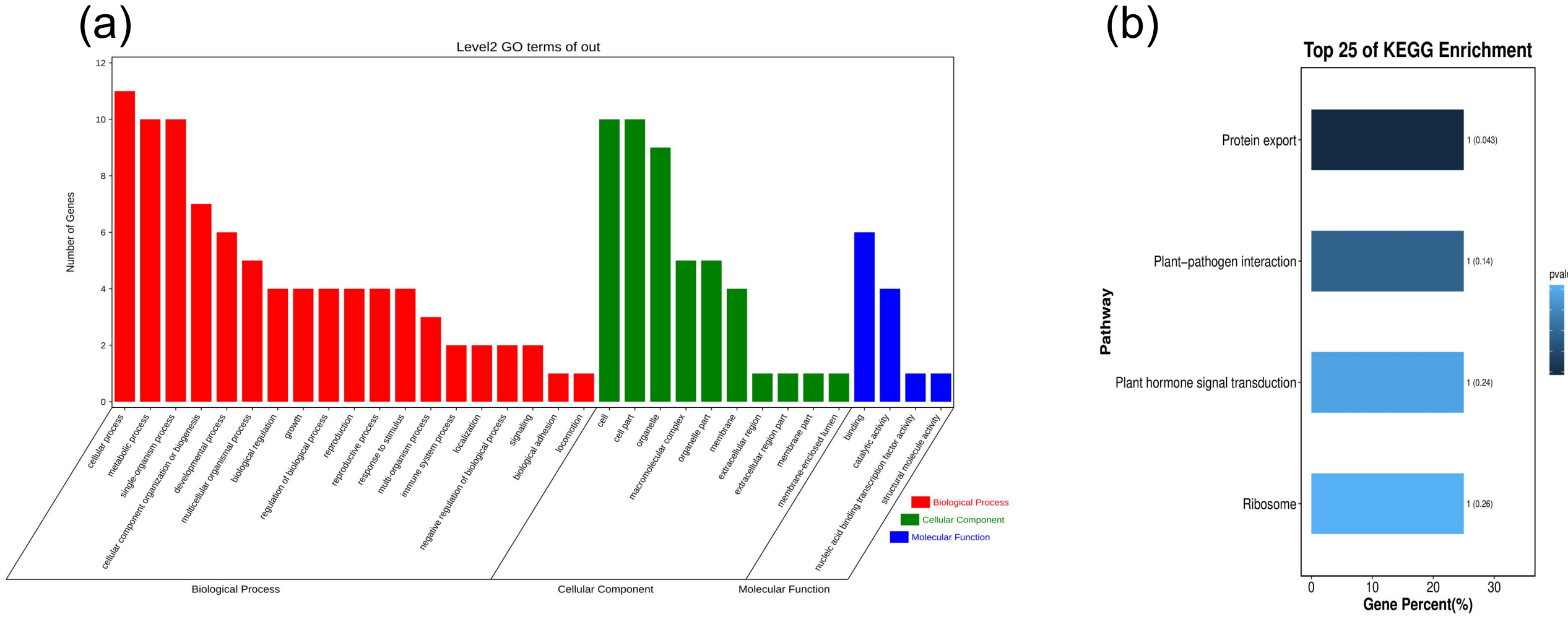
(a)
Level2 GO terms of out
Number of Genes
0
2
4
6
8
10
12
cellular process
metabolic process
single-organism process
cellular component organization or biogenesis
developmental process
multicellular organismal process
biological regulation
growth
regulation of biological process
reproduction
reproductive process
response to stimulus
multi-organism process
immune system process
localization
negative regulation of biological process
signaling
biological adhesion
locomotion
cell
cell part
organelle
macromolecular complex
organelle part
membrane
extracellular region
extracellular region part
membrane part
membrane-enclosed lumen
binding
catalytic activity
nucleic acid binding transcription factor activity
structural molecule activity
Biological Process
Cellular Component
Molecular Function
Biological Process
Cellular Component
Molecular Function
(b)
Top 25 of KEGG Enrichment
Pathway
Protein export
Plant−pathogen interaction
Plant hormone signal transduction
Ribosome
1 (0.043)
1 (0.14)
1 (0.24)
1 (0.26)
0
10
20
30
Gene Percent(%)
pvalue
0.25
0.20
0.15
0.10
0.05

Supplementary Figure 1. Functional classification of the candidate genes within the GWAS significant locus. (a) Gene Ontology (GO) classification analysis of the 30 annotated candidate genes located within the linkage disequilibrium (LD) block on Ft8. The results indicate that the majority of these genes are concentrated in cellular and metabolic processes. (b) KEGG pathway classification of candidate genes. The genes are primarily include in immune-related pathways, specifically "plant-pathogen interaction" and "plant hormone signal transduction"

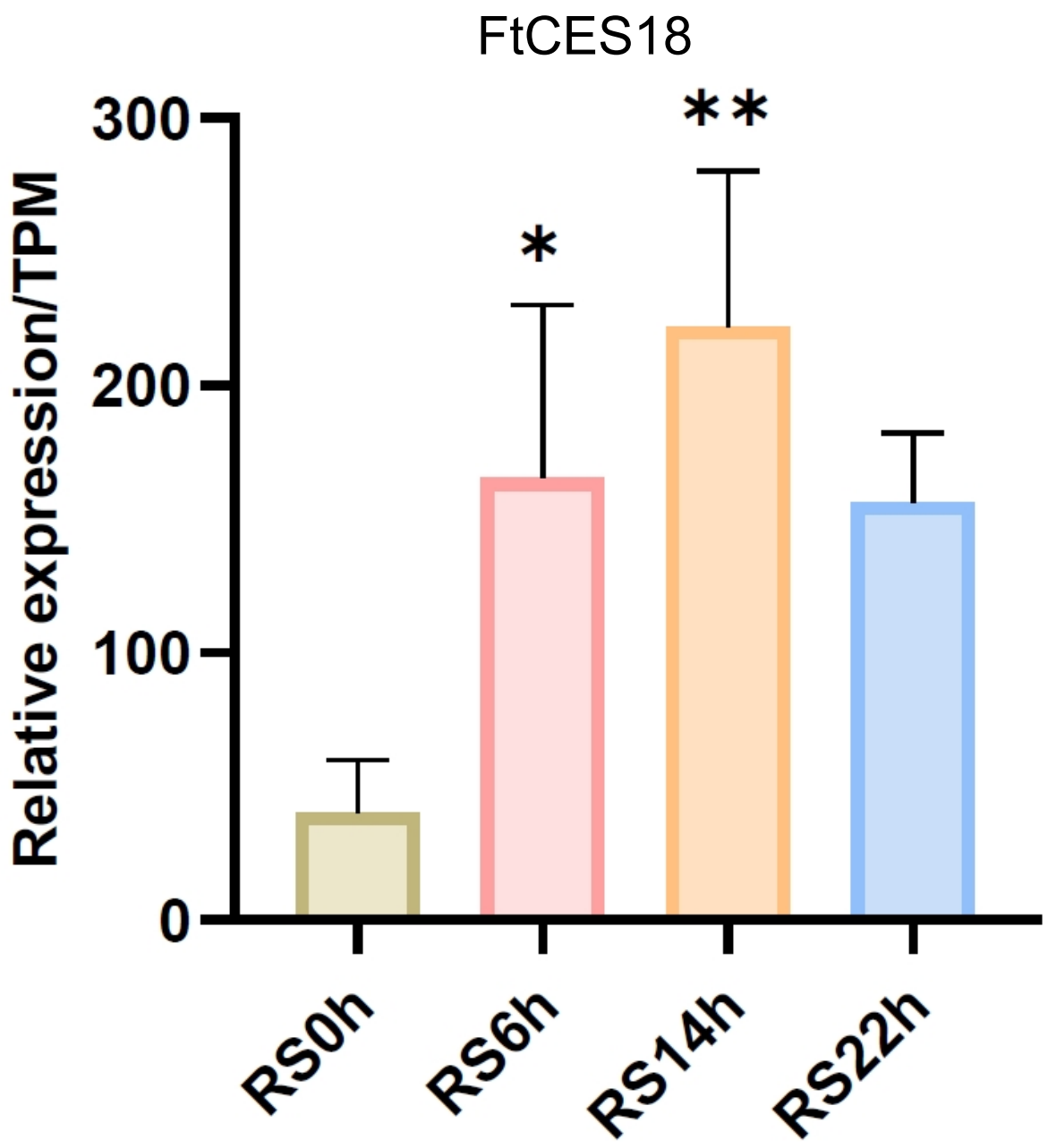
FtCES18
Relative expression/TPM
300
200
100
0
*
**
RS0h
RS6h
RS14h
RS22h

Supplementary Figure 2. Expression profile of *FtCES18* in response to *R. solani* infection. The temporal expression levels of *FtCES18* in Tartary buckwheat at different time points post-inoculation with *R. solani*, as derived from the interaction transcriptome data (*P < 0.05, **P < 0.01; Student's t-test)..

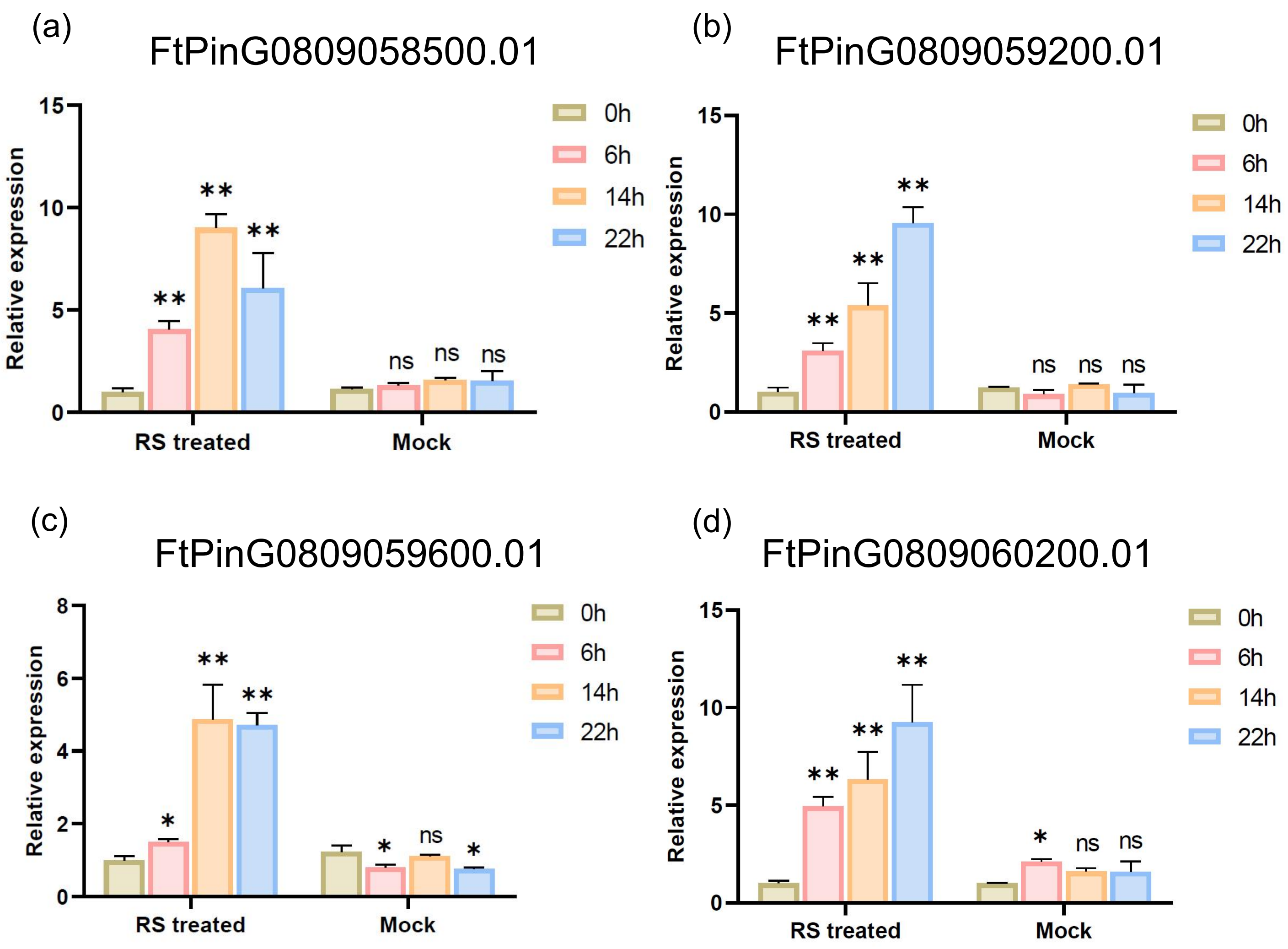
(a)
FtPinG0809058500.01
(b)
FtPinG0809059200.01
(c)
FtPinG0809059600.01
(d)
FtPinG0809060200.01
Relative expression
RS treated
Mock
0h
6h
14h
22h
**
*
ns

Supplementary Figure 3. Time-course expression profiles of four candidate genes under *R. solani* challenge and mock conditions. (a–d) Relative transcript abundance of four candidate genes—(a) *FtPinG0809058500.01*, (b) *FtPinG0809059200.01*, (c) *FtPinG0809059600.01*, and (d) *FtPinG0809060200.01*—in Tartary buckwheat leaves inoculated with *R. solani* (RS treated) or blank agar plugs (Mock) across 0, 6, 14, and 22 h post-inoculation (hpi), as quantified by RT-qPCR. Expression levels of each gene at 0 h were set to 1.0 for normalization. Data are presented as mean ± SD of three independent biological replicates. Asterisks denote statistically significant differences compared with the corresponding 0 h control (*$P < 0.05$, **$P < 0.01$; ns, not significant; Student's t-test).

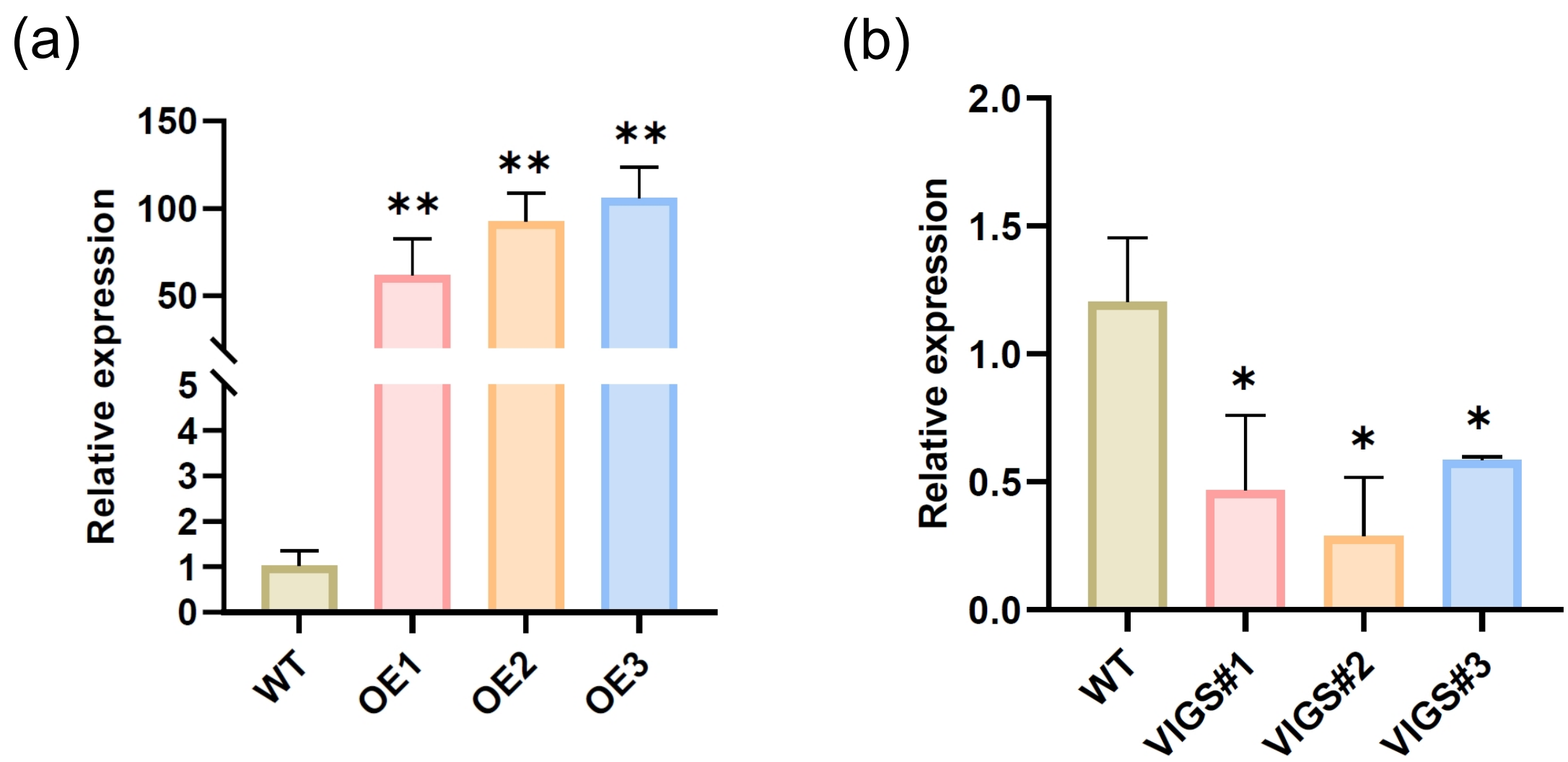
(a)
150
100
50
5
4
3
2
1
0
Relative expression
**
**
**
WT
OE1
OE2
OE3
(b)
2.0
1.5
1.0
0.5
0.0
Relative expression
*
*
*
WT
VIGS#1
VIGS#2
VIGS#3

Supplementary Figure 4. Relative expression levels of *FtCES18* in different transgenic lines. (a) Relative expression levels of *FtCES18* in wild-type (WT) and three independent *FtCES18*-overexpressing (OE1, OE2, OE3) *Arabidopsis* lines. (b) Relative expression levels of *FtCES18* in the Mock control and *FtCES18*-silenced (VIGS#1, VIGS#2, VIGS#3) Tartary buckwheat leaves. Data are presented as means ± SD from three independent biological replicates (*P < 0.05, **P < 0.01; Student's t-test).

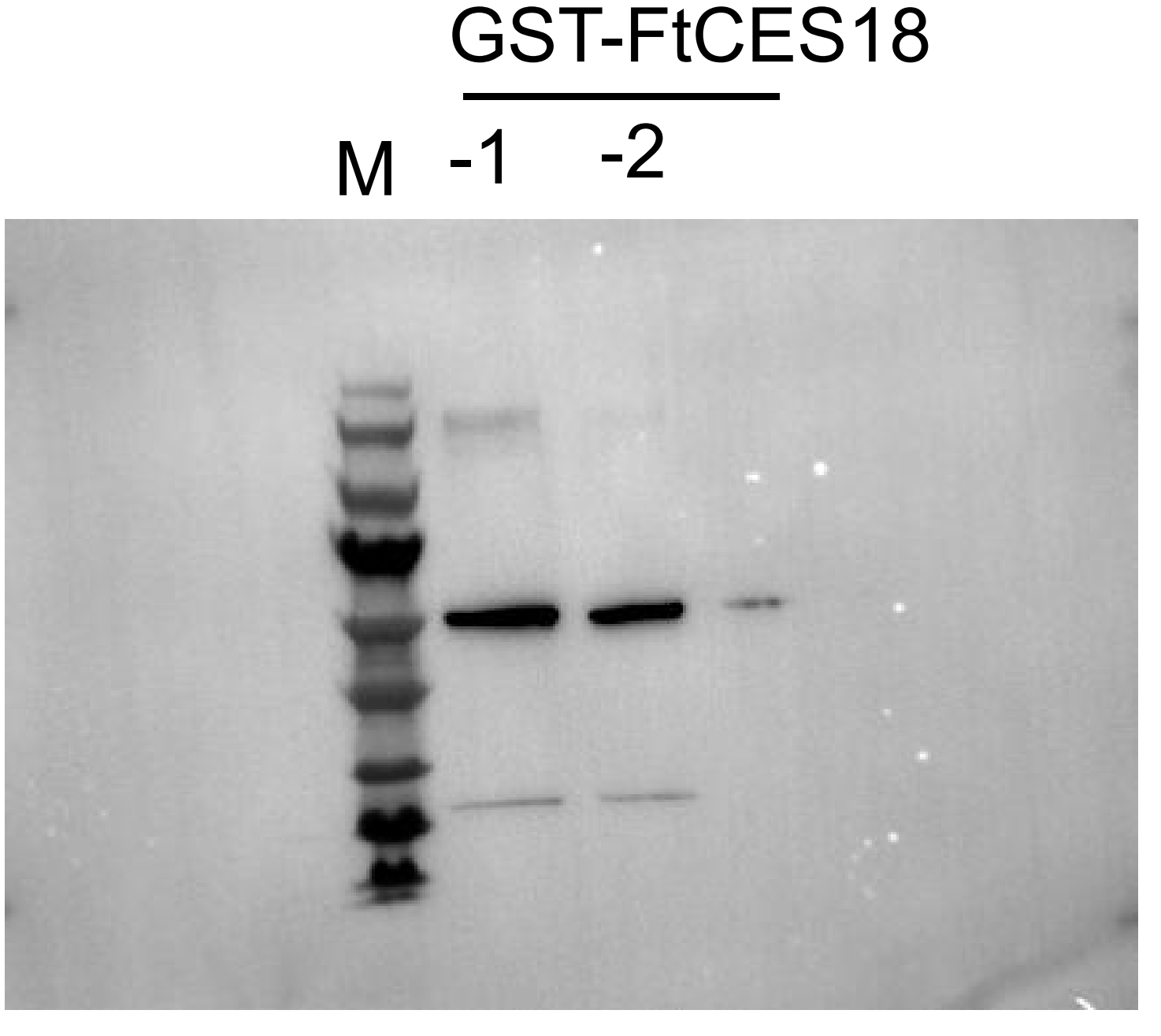
GST-FtCES18
M
-1
-2

Supplementary Figure 5. Western blot analysis of purified GST-FtCES18 protein. M: M5 Prestained Protein Ladder(10-180 kDa).

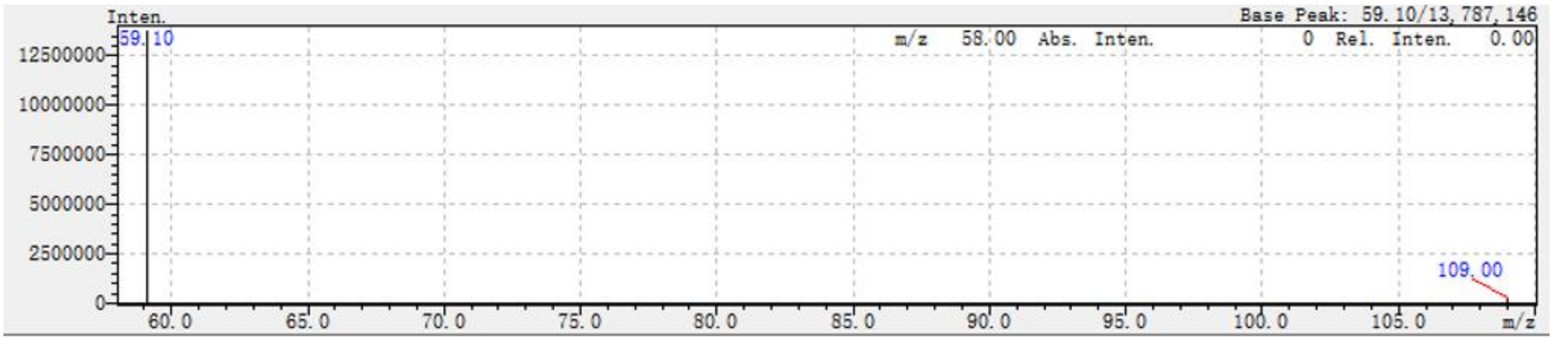
Inten.
Base Peak: 59.10/13,787,146
m/z 58.00 Abs. Inten. 0 Rel. Inten. 0.00
59.10
109.00
12500000
10000000
7500000
5000000
2500000
0
60.0
65.0
70.0
75.0
80.0
85.0
90.0
95.0
100.0
105.0
m/z

Supplementary Figure 6. LC-MS/MS product ion spectrum of the enzymatic reaction product generated by recombinant FtCES18. Representative MS/MS spectrum (product ion scan) of the precursor ion at m/z 209.15 ([M-H]- of JA) acquired under negative electrospray ionization (ESI-) mode with a collision energy of 25.0 eV. The characteristic product ions at m/z 59.05 (base peak) and m/z 109.00 confirm the structural identity of the reaction product as free JA.

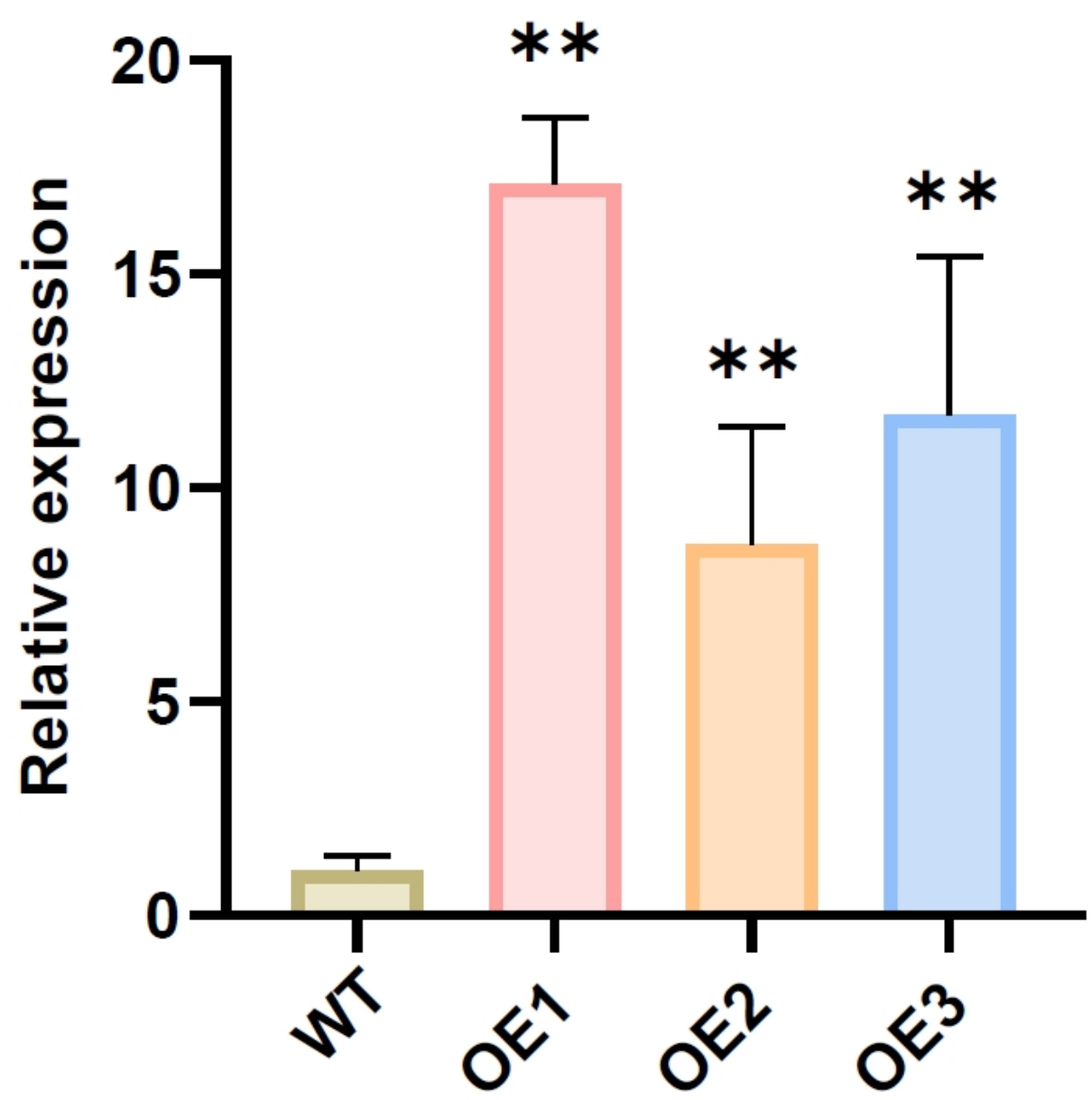
Relative expression
20
15
10
5
0
**
**
**
WT
OE1
OE2
OE3

Supplementary Figure 7. Relative expression levels of *FtCES18* in transgenic hairy roots. Relative expression levels of *FtCES18* in wild-type and three independent *FtCES18*-overexpressing (OE1, OE2, OE3) Tartary buckwheat hairy root lines. Data are presented as means ± SD from three independent biological replicates(*P < 0.05, **P < 0.01; Student's t-test)..

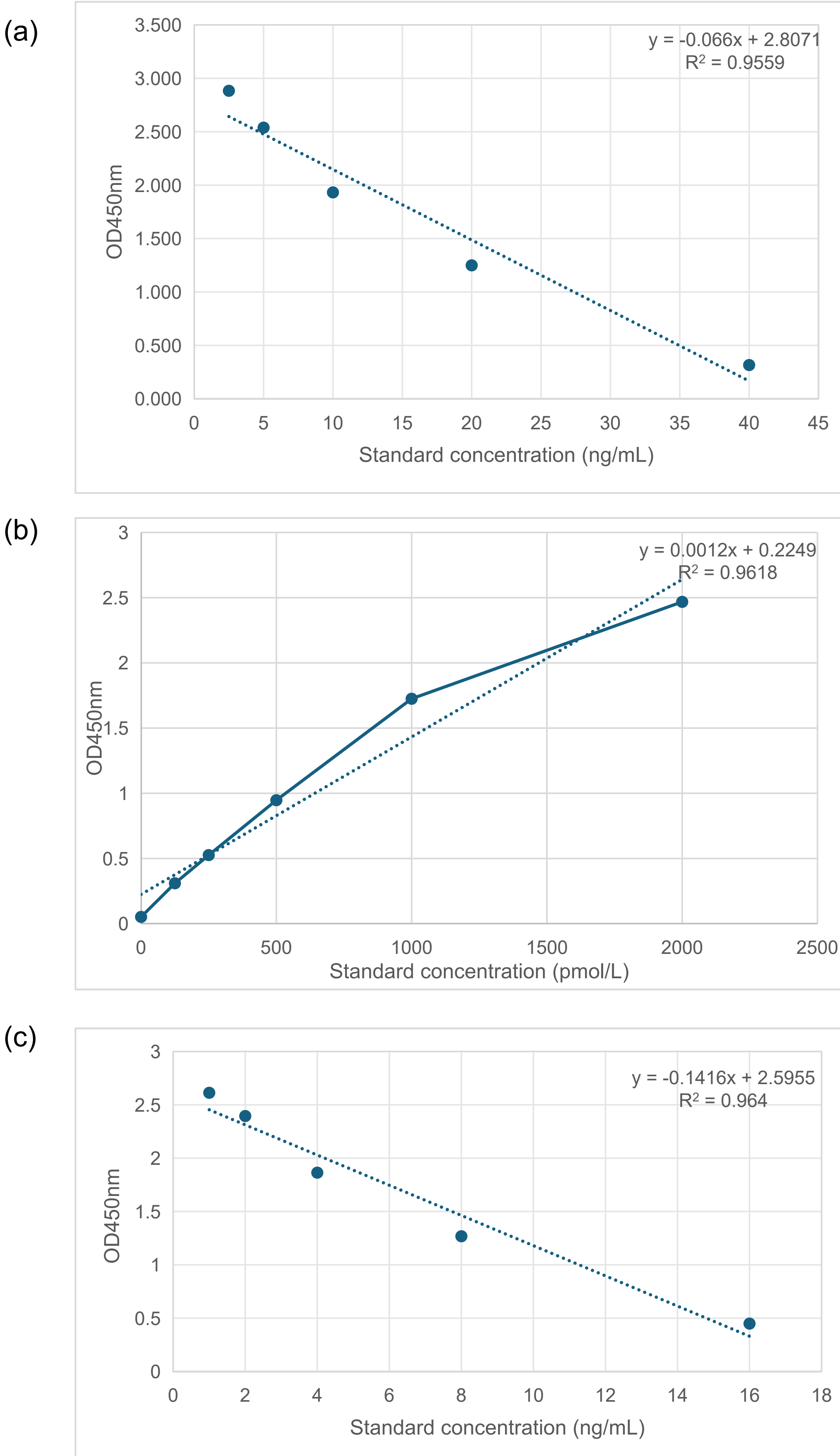
(a)
y = -0.066x + 2.8071
R² = 0.9559
OD450nm
Standard concentration (ng/mL)
(b)
y = 0.0012x + 0.2249
R² = 0.9618
OD450nm
Standard concentration (pmol/L)
(c)
y = -0.1416x + 2.5955
R² = 0.964
OD450nm
Standard concentration (ng/mL)

Supplementary Figure 8. Quantitative standard curves for MeJA (a), JA (b), and JA-Ile (c). These standard curves were generated using standard samples from an enzyme-linked immunosorbent assay (ELISA) kit.

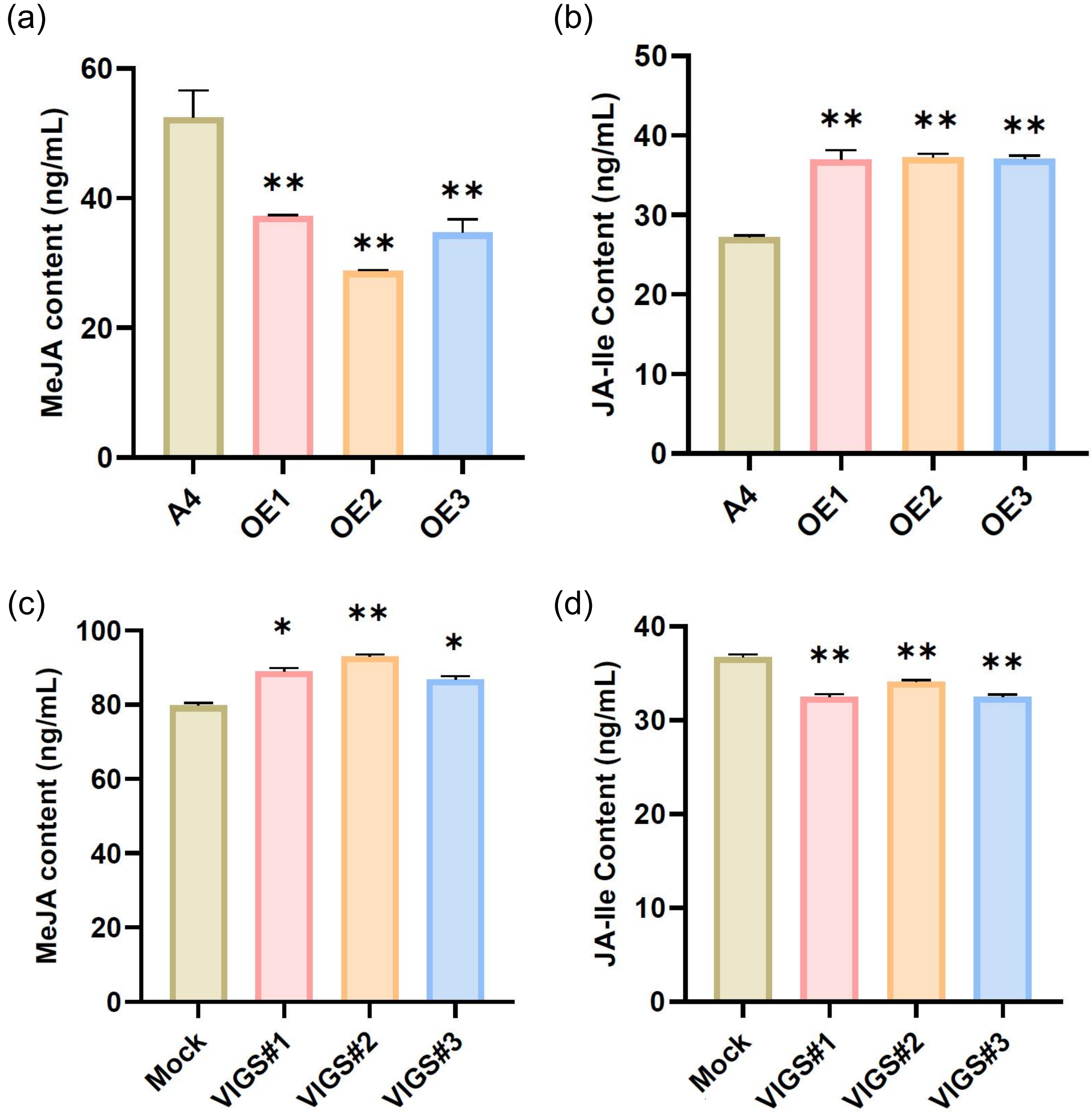
(a)
MeJA content (ng/mL)
60
40
20
0
A4
OE1
OE2
OE3
**
**
**
(b)
JA-Ile Content (ng/mL)
50
40
30
20
10
0
A4
OE1
OE2
OE3
**
**
**
(c)
MeJA content (ng/mL)
100
80
60
40
20
0
Mock
VIGS#1
VIGS#2
VIGS#3
*
**
*
(d)
JA-Ile Content (ng/mL)
40
30
20
10
0
Mock
VIGS#1
VIGS#2
VIGS#3
**
**
**

Supplementary Figure 9. Endogenous MeJA and JA-Ile contents in *FtCES18*-overexpressing and *FtCES18*-silenced Tartary buckwheat lines. (a) Endogenous MeJA content and (b) JA-Ile content in wild-type control (A4) and three independent *FtCES18*-overexpressing Tartary buckwheat hairy root lines. (c) Endogenous MeJA content and (d) JA-Ile content in control (Mock) and three independent *FtCES18*-silenced Tartary buckwheat leaves. Data in bar graphs are presented as mean ± SD of three independent biological replicates. Asterisks denote statistically significant differences compared with the respective controls (A4 for hairy roots, Mock for VIGS leaves) (*P < 0.05, **P < 0.01; Student's t-test).

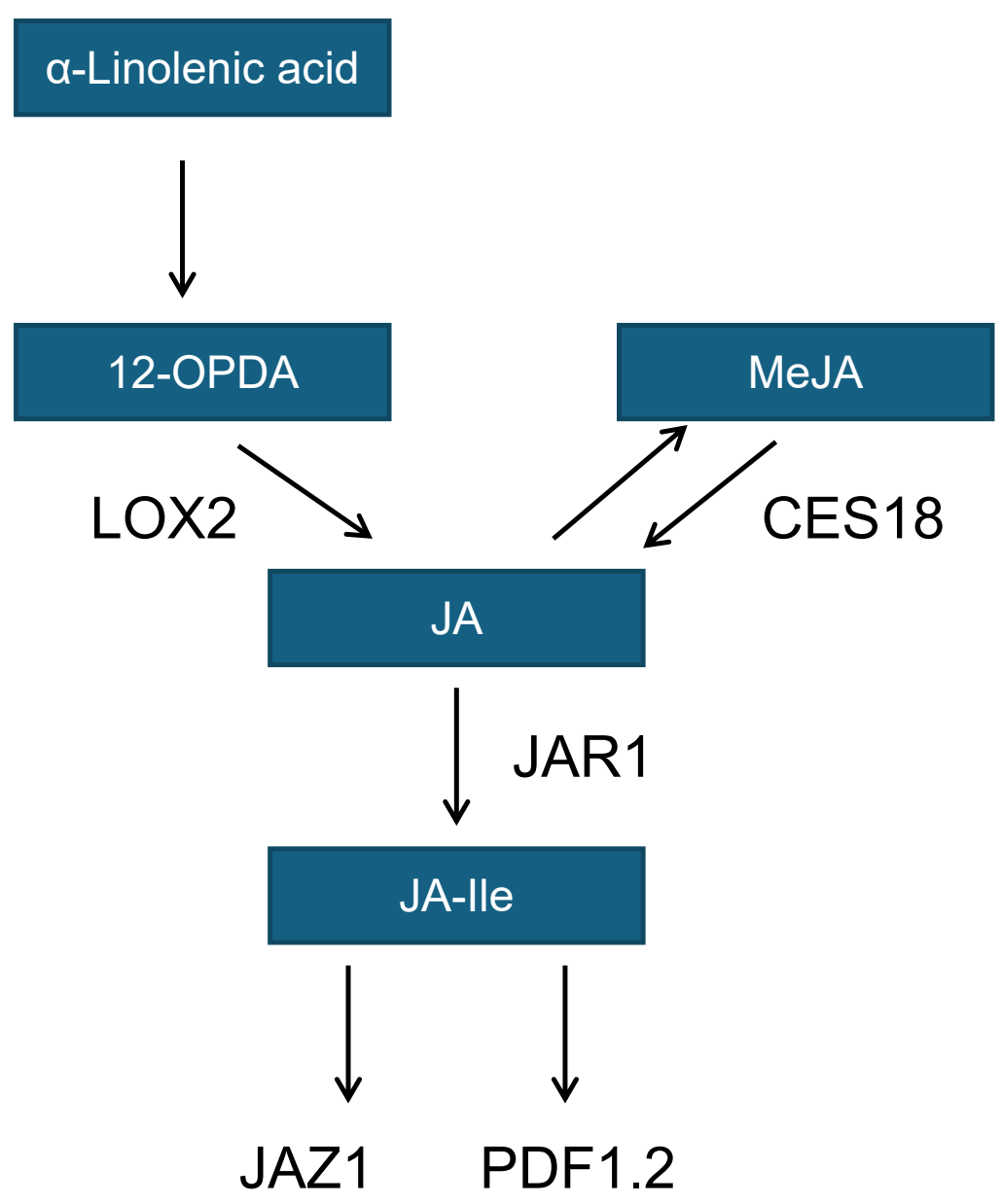
α-Linolenic acid
12-OPDA
MeJA
LOX2
CES18
JA
JAR1
JA-Ile
JAZ1
PDF1.2

Supplementary Figure 10. A simplified schematic of the JA biosynthesis and signaling pathway. The diagram illustrates the conversion of key metabolites and the specific steps catalyzed by the genes evaluated in this study, including *AtLOX2*, *FtCES18*, *AtJAR1*, and the downstream responsive genes *AtJAZ1* and *AtPDF1.2*. 12-OPDA, 12-oxophytodienoic acid; MeJA. Methyl jasmonate; JA-Ile, Jasmonoyl-isoleucine; *LOX2*, Lipoxygenase; *JAR1*, Jasmonate Resistant 1; *JAZ1*, Jasmonate ZIM-domain protein; *PDF1.2*, Plant Defensin 1.2.

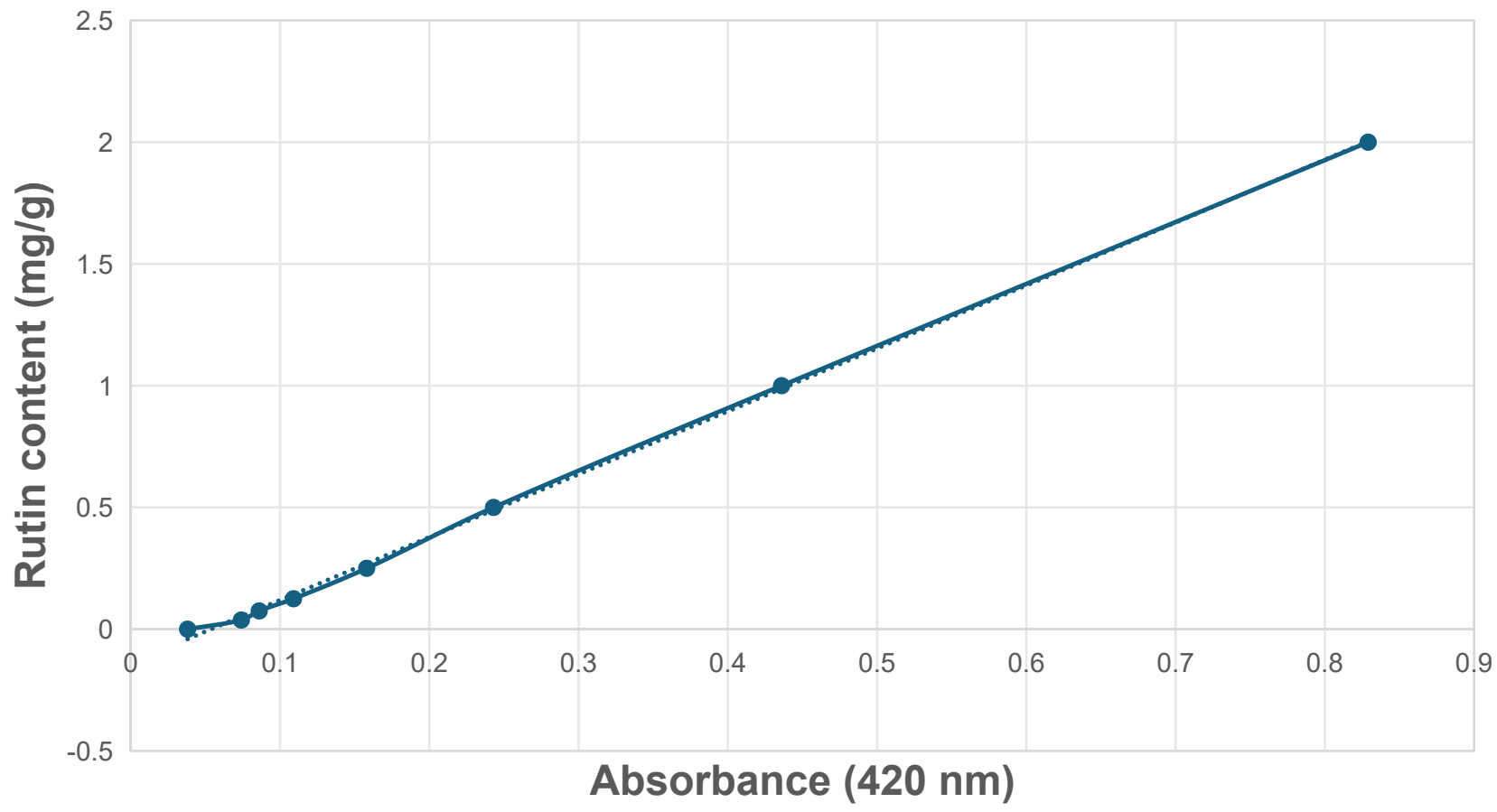

Rutin content (mg/g)
Absorbance (420 nm)
2.5
2
1.5
1
0.5
0
-0.5
0
0.1
0.2
0.3
0.4
0.5
0.6
0.7
0.8
0.9

Supplementary Figure 11. Standard curve for total flavonoid quantification. The standard curve was generated to determine the total flavonoid concentrations in the samples. The linear regression equation of the standard curve is $y = 2.5839x - 0.1388$ ($R^2 = 0.9991$).

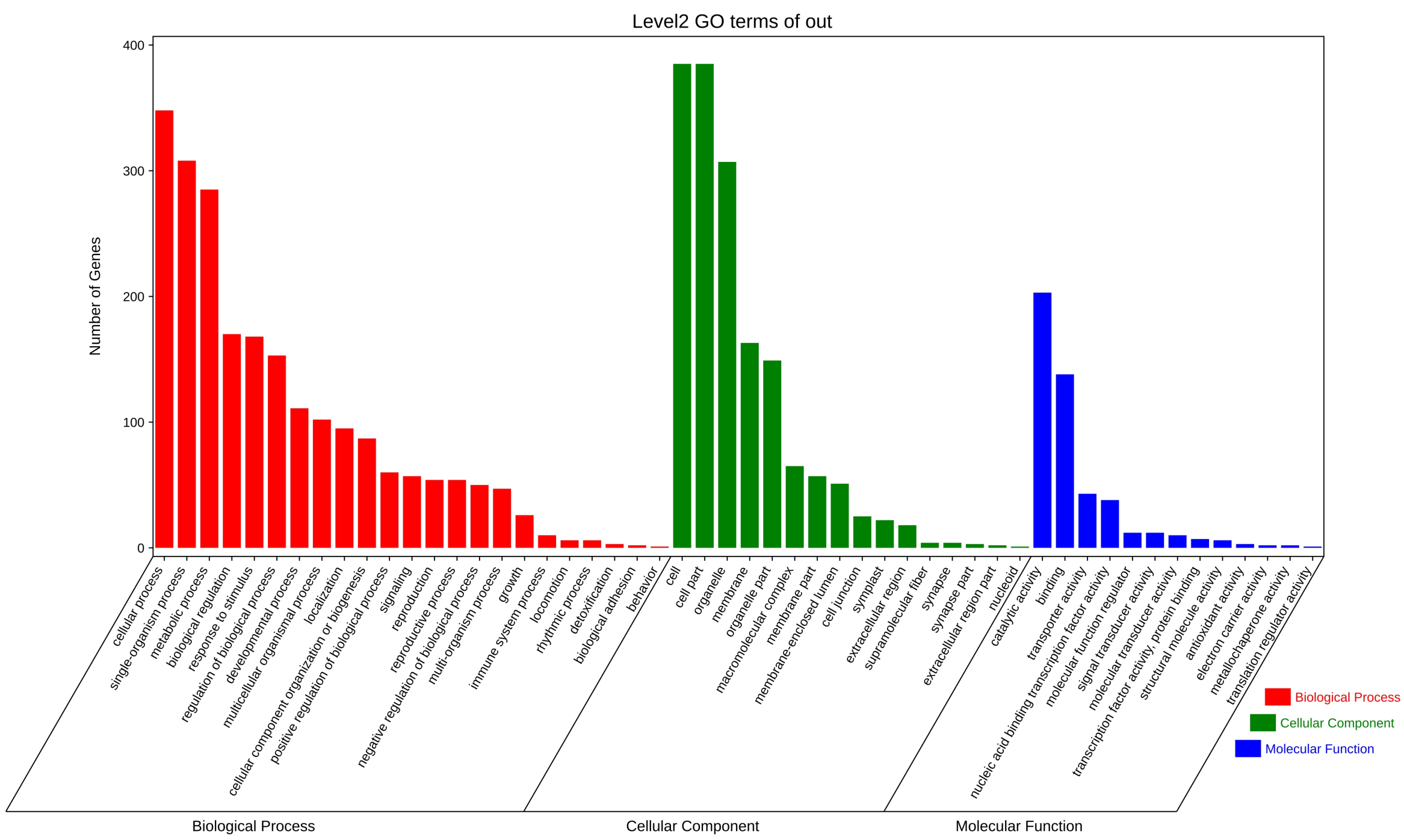

Level2 GO terms of out
Number of Genes
0
100
200
300
400
cellular process
single-organism process
metabolic process
biological regulation
response to stimulus
regulation of biological process
developmental process
multicellular organismal process
localization
cellular component organization or biogenesis
positive regulation of biological process
signaling
reproduction
reproductive process
negative regulation of biological process
multi-organism process
growth
immune system process
locomotion
rhythmic process
detoxification
biological adhesion
behavior
cell
cell part
organelle
membrane
organelle part
macromolecular complex
membrane part
membrane-enclosed lumen
cell junction
symplast
extracellular region
supramolecular fiber
synapse
synapse part
extracellular region part
nucleoid
catalytic activity
binding
transporter activity
nucleic acid binding transcription factor activity
molecular function regulator
signal transducer activity
molecular transducer activity
transcription factor activity, protein binding
structural molecule activity
antioxidant activity
electron carrier activity
metallochaperone activity
translation regulator activity
Biological Process
Cellular Component
Molecular Function
Biological Process
Cellular Component
Molecular Function

Supplementary Figure 12. Gene Ontology (GO) classification of co-expressed genes in Cluster 8. The analysis reveals that the genes within the *R. solani*-induced Cluster 8 module are broadly involved in core biological processes, including "metabolic process" and "response to stimulus".

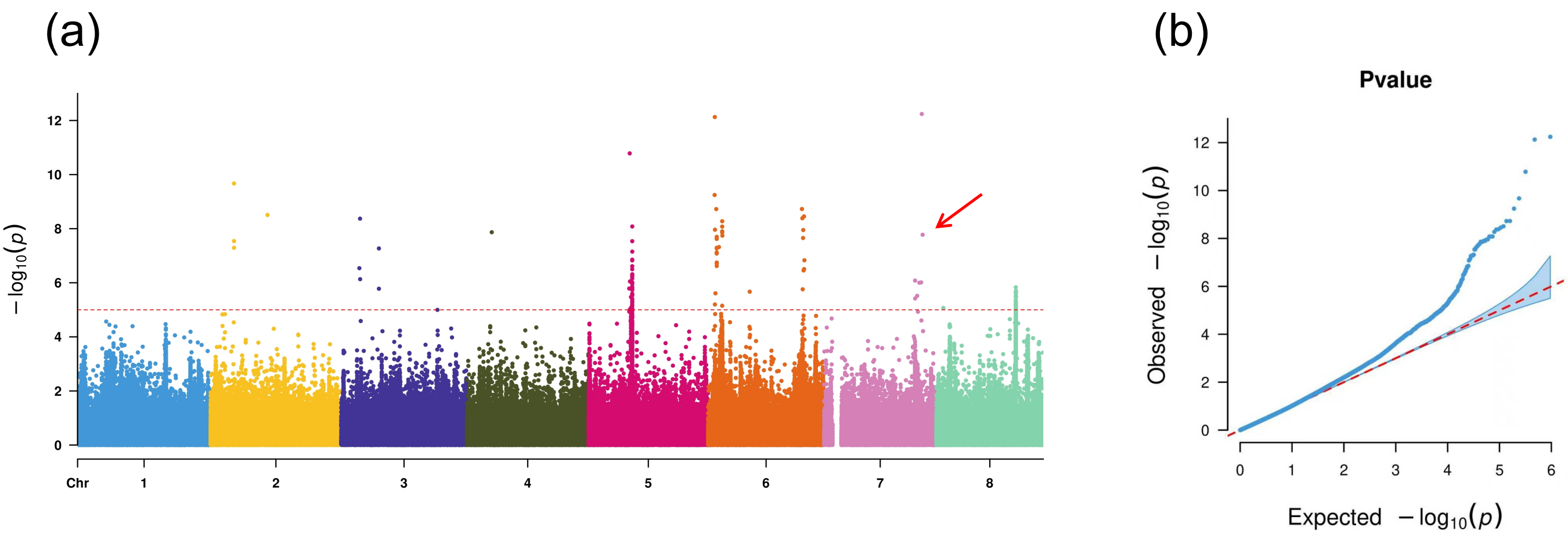

(a)
12
10
8
6
4
2
0
−log10(p)
Chr
1
2
3
4
5
6
7
8
(b)
Pvalue
Observed −log10(p)
Expected −log10(p)

Supplementary Figure 13. GWAS signals for quercetin content trait. (a) Manhattan plot displaying the significant SNP signals associated with quercetin content. The red arrow indicates the genomic locus containing *FtMYB32*. The red dashed line represents the genome-wide significance threshold. (b) Quantile-quantile (Q-Q) plot for the GWAS of quercetin content.

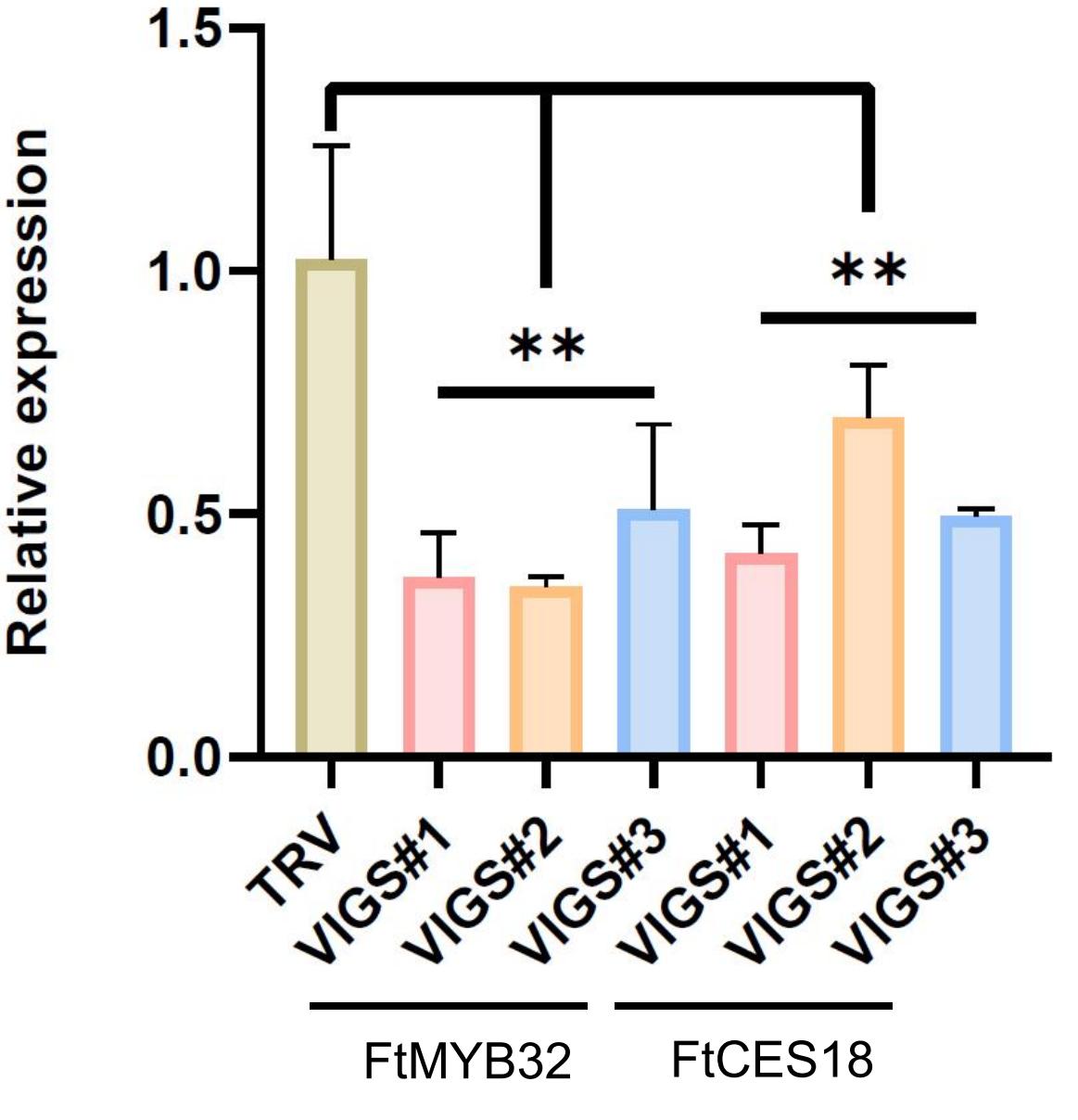
1.5
1.0
0.5
0.0
Relative expression
**
**
TRV
VIGS#1
VIGS#2
VIGS#3
VIGS#1
VIGS#2
VIGS#3
FtMYB32
FtCES18

Supplementary Figure 14. Relative transcript abundance of *FtMYB32* and downstream *FtCES18* in control (TRV) and three independent *FtMYB32*-silenced (*FtMYB32*-VIGS) Tartary buckwheat leaves. Asterisks denote statistically significant differences compared with the TRV control (Student's t-test; $^{**}P < 0.01$).

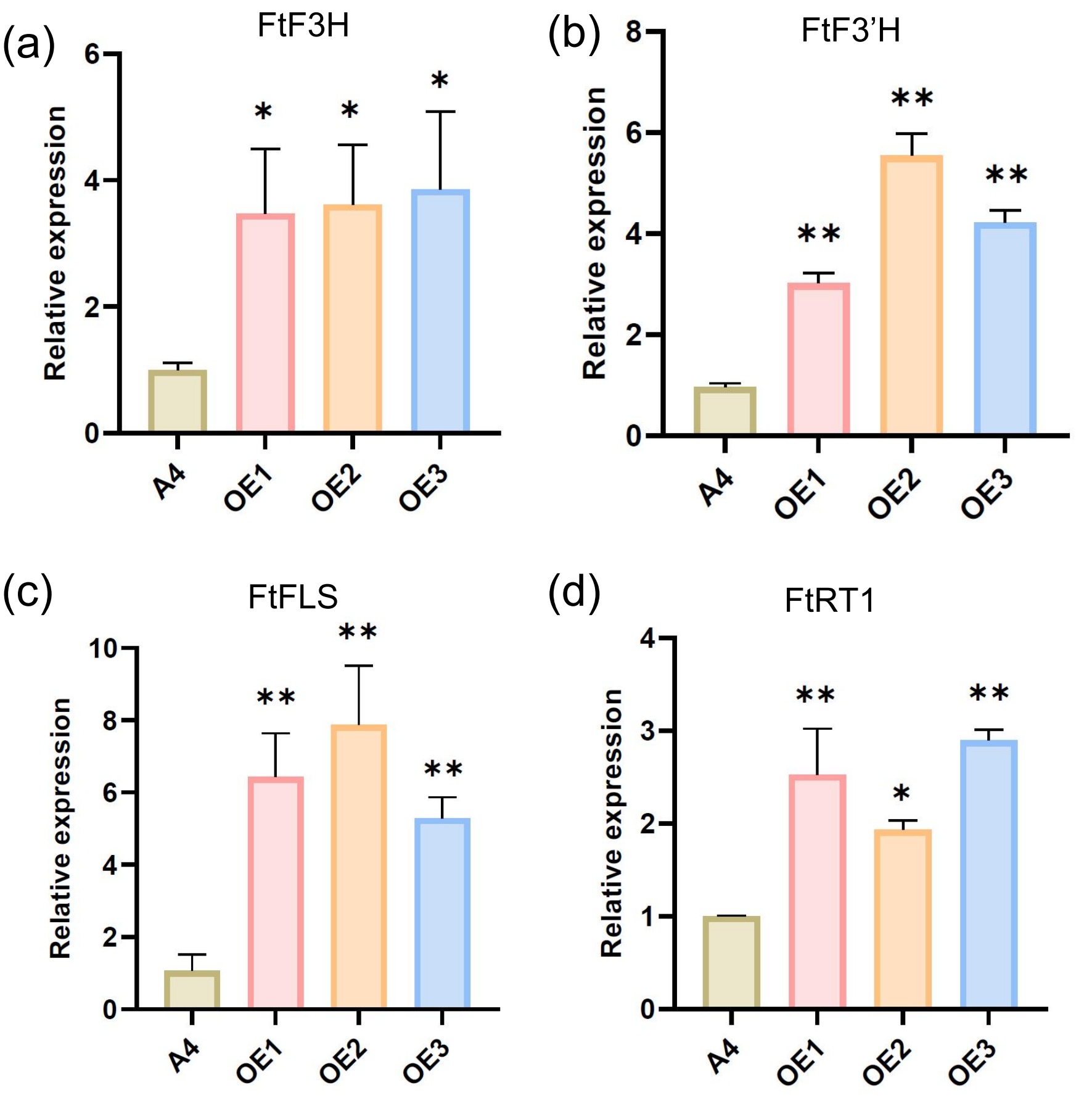

(a)
FtF3H
Relative expression
A4
OE1
OE2
OE3
(b)
FtF3'H
Relative expression
A4
OE1
OE2
OE3
(c)
FtFLS
Relative expression
A4
OE1
OE2
OE3
(d)
FtRT1
Relative expression
A4
OE1
OE2
OE3

Supplementary Figure 15. Relative transcript abundance of key flavonoid biosynthetic structural genes, including flavanone 3-hydroxylase (*FtF3H*) (a), flavonoid 3'-hydroxylase (*FtF3'H*) (b), flavonol synthase (*FtFLS*) (c), and rutin rhamnosyltransferase 1 (*FtRT1*) (d), in control (A4) and three independent *FtMYB32*-overexpressing (*FtMYB32*-OE) Tartary buckwheat hairy root lines. Data are presented as mean ± SD of three independent biological replicates. Asterisks denote statistically significant differences compared with the A4 control (*$P < 0.05$, **$P < 0.01$; Student's-test).

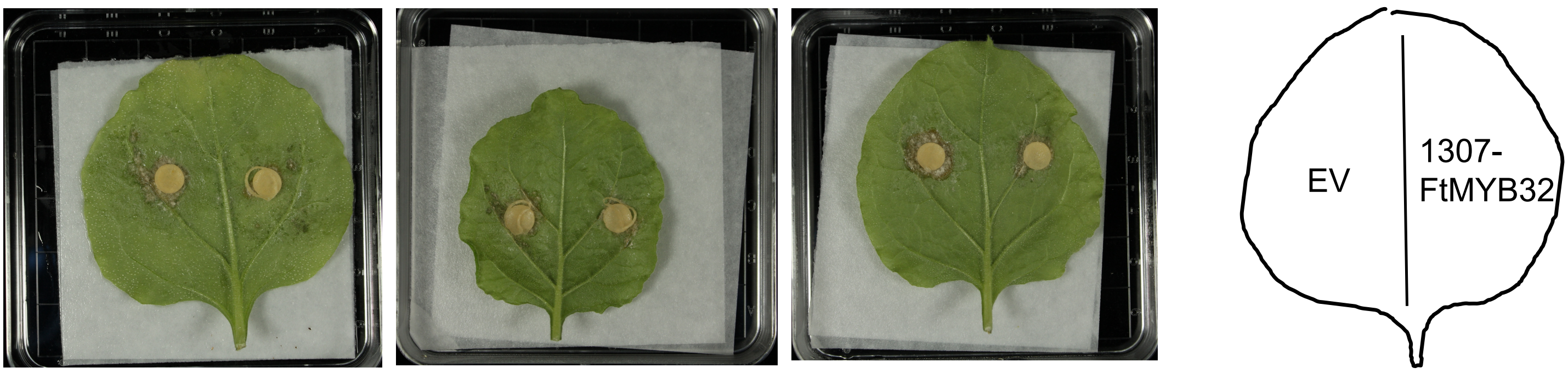
EV
1307-
FtMYB32

Supplementary Figure 16. Transient expression of *FtMYB32* enhances resistance to *R. solani* in *N. benthamiana* leaves. The left half of each leaf was agroinfiltrated with an empty vector (EV) control, while the right half was infiltrated with the 1307-*FtMYB32* overexpression construct.

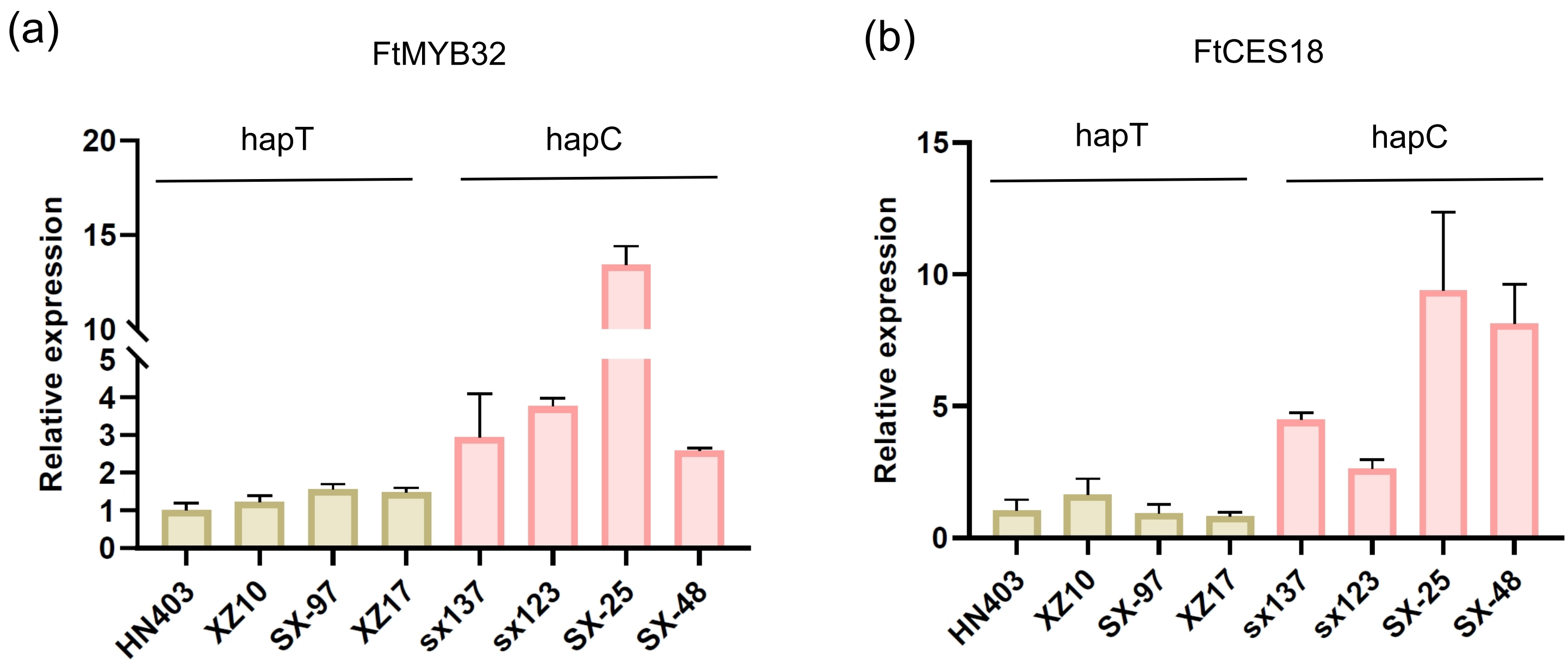

(a)
FtMYB32
hapT
hapC
Relative expression
20
15
10
5
4
3
2
1
0
HN403
XZ10
SX-97
XZ17
sx137
sx123
SX-25
SX-48
(b)
FtCES18
hapT
hapC
Relative expression
15
10
5
0
HN403
XZ10
SX-97
XZ17
sx137
sx123
SX-25
SX-48

Supplementary Figure 17. Relative expression levels of *FtMYB32* and *FtCES18* in Tartary buckwheat accessions with different haplotypes. (a) Relative expression levels of *FtMYB32* in representative accessions carrying the hapT or hapC haplotype. (b) Relative expression levels of *FtCES18* in the corresponding accessions. Data are presented as means ± SD from three independent biological replicates.
